# Critical Evaluation of P2X7 Receptor Antagonists in Selected Seizure Models

**DOI:** 10.1371/journal.pone.0156468

**Published:** 2016-06-09

**Authors:** Wolfgang Fischer, Heike Franke, Ute Krügel, Heiko Müller, Klaus Dinkel, Brian Lord, Michael A. Letavic, David C. Henshall, Tobias Engel

**Affiliations:** 1 Rudolf-Boehm-Institute of Pharmacology and Toxicology, Medical Faculty, University of Leipzig, Leipzig, Germany; 2 Lead Discovery Center GmbH, Dortmund, Germany; 3 Affectis Pharmaceutical AG, Dortmund, Germany; 4 Neuroscience Therapeutic Area, Janssen Research & Development, LLC, San Diego, California, United States of America; 5 Department of Physiology and Medical Physics, Royal College of Surgeons in Ireland, Dublin 2, Ireland; University Paris Sud, FRANCE

## Abstract

The ATP-gated P2X7 receptor (P2X7R) is a non-selective cation channel which senses high extracellular ATP concentrations and has been suggested as a target for the treatment of neuroinflammation and neurodegenerative diseases. The use of P2X7R antagonists may therefore be a viable approach for treating CNS pathologies, including epileptic disorders. Recent studies showed anticonvulsant potential of P2X7R antagonists in certain animal models. To extend this work, we tested three CNS-permeable P2X7R blocker (Brilliant Blue G, AFC-5128, JNJ-47965567) and a natural compound derivative (tanshinone IIA sulfonate) in four well-characterized animal seizure models. In the maximal electroshock seizure threshold test and the pentylenetetrazol (PTZ) seizure threshold test in mice, none of the four compounds demonstrated anticonvulsant effects when given alone. Notably, in combination with carbamazepine, both AFC-5128 and JNJ-47965567 increased the threshold in the maximal electroshock seizure test. In the PTZ-kindling model in rats, useful for testing antiepileptogenic activities, Brilliant Blue G and tanshinone exhibited a moderate retarding effect, whereas the potent P2X7R blocker AFC-5128 and JNJ-47965567 showed a significant and long-lasting delay in kindling development. In fully kindled rats, the investigated compounds revealed modest effects to reduce the mean seizure stage. Furthermore, AFC-5128- and JNJ-47965567-treated animals displayed strongly reduced Iba 1 and GFAP immunoreactivity in the hippocampal CA3 region. In summary, our results show that P2X7R antagonists possess no remarkable anticonvulsant effects in the used acute screening tests, but can attenuate chemically-induced kindling. Further studies would be of interest to support the concept that P2X7R signalling plays a crucial role in the pathogenesis of epileptic disorders.

## Introduction

Epilepsy comprises a family of chronic neurological diseases with different causes and symptoms characterized by an enduring state of spontaneous and recurrent seizure activity. Patients with epilepsy experience different types of seizures. Most seizures can be categorized as either partial (e.g., simple and complex partial seizures depending on retained awareness or loss) or generalized (e.g., generalized tonic-clonic, clonic, myoclonic, absence and atonic seizures). Epilepsy affects about 1% of the population worldwide and represents a serious global public health issue [1–3]. The underlying pathophysiological mechanisms are still incompletely understood and present treatment is dominated by pharmacotherapy with anticonvulsant drugs (antiepileptic drugs; AEDs). Current AEDs provide symptomatic relief of seizures, but without any influence on the course of disease. Despite the introduction of various new AEDs in recent years, about a third of patients remain resistant to current pharmacotherapies [4]. Furthermore, there is no effective treatment to prevent the genesis or progression of seizures or the development of drug-resistant epilepsy [5, 6]. Thus, new insights in the cellular and molecular mechanisms of epilepsy would offer novel targets for the development of innovative therapeutic strategies [7, 8].

Increasing evidence points to the involvement of brain inflammation, linked with glial cell activation and the release of pro-inflammatory mediators including interleukins, tumour necrosis factor alpha (TNF-α) as well as a variety of other cytokines, in the pathogeneses of many neurological disorders, including epilepsy [9–12]. In particular, activation of the pro-inflammatory axis formed by interleukin-1ß (IL-1β) and its cognate receptor IL-1R type 1 has been described in patients with epilepsy as well as in experimental models of epilepsy [13–17]. These data support future design and testing of antiepileptic strategies affecting specific pro-inflammatory pathways such as the IL-1β-related signalling cascade (for review, see [18]).

In this regard, the ATP-gated purinergic P2X7 receptor (P2X7R) may represent a novel target for interruption of neuroinflammation, seizure control and neuroprotection [19–21]. The P2X7R is a membrane-bound, non-selective cation channel, which senses high extracellular ATP concentrations and activates a particularly wide range of signal transduction pathways. This receptor seems to be the most divergent member of the ionotropic purinergic P2X receptor family in terms of its structure, pharmacology and function [22–24]. In the brain, the P2X7R is primarily expressed in microglia and ependymal cells, and to a lesser extent in oligodendrocytes, astrocytes, and possibly, in some populations of neurons as well as presynaptic terminals; however, some findings remain controversial [25–28]. Receptor activation triggers not only trans-membrane ion fluxes (Ca^2+^/Na^+^; K^+^) that mediate fast excitatory transmission, but is also involved in the release of glutamate, GABA and ATP from neuronal terminals and astrocytes. In particular, the P2X7R is a major regulator of the activation of the inflammasome complex and cytokine IL-1β secretion by glial cells, which may play a pivotal role in inflammatory processes [29–32]. Furthermore, the channel activity is poorly desensitising, and prolonged exposure to ATP may result in the formation of a transmembrane pore, an event associated with cytotoxic effects [33, 34]. P2X7R-deficient mice showed an attenuation of the release of pro-inflammatory cytokines [35] and substantially attenuated inflammatory responses, linked with reduced inflammatory and neuropathic pain sensitivity [36–38].

The P2X7R may also represent a target for disease-modification in epilepsy. It is well-established that brain injury, e.g. neurotrauma, stroke, ischemia or prolonged febrile seizures, represents an important risk factor for the development of epilepsy [39, 40]. Brain injury is thought to lead to the release of high concentrations of ATP as a consequence of lesion and cell death that acts as a danger signal through the activation of P2X7R and possibly additional purinergic P2 receptors [41–43]. High extracellular ATP and continuing brain inflammation via P2X7R-mediated gliosis and increased cytokine production in turn promotes neuronal hyper-excitability through modifications of various ion channels, changes in transmitter release and damage of the blood-brain barrier [6, 7, 12]. This self-amplifying cascade is mediated predominantly by the pro-inflammatory cytokine IL-1β, which worsened seizure activity after intra-hippocampal injection. Conversely, IL-1β biosynthesis inhibition or the IL-1 receptor antagonist IL-1Ra showed anticonvulsant activity in various seizure models [44, 45]. As a key mediator of the release of IL-1β, the P2X7R may function as an important interface between brain inflammation and epileptogenesis. Experimental data showed that P2X7R expression is upregulated in microglia and/or neurons after status epilepticus and in chronic epileptic animals [46–51]. Increased P2X7R levels also have been documented in the hippocampus or neocortex of patients with temporal lobe epilepsy [49, 51–53]. Consequently, it is suggested that P2X7R antagonism may be a viable therapeutic approach for treating epilepsy and also other neurological diseases [20, 54, 55].

To date, the vast majority of studies to elucidate the role of P2X7R activation during seizures has been carried out in mouse models of status epilepticus using kainic acid (KA) or pilocarpine as chemoconvulsant [21, 50]. Recent data provide support for the use of P2X7R antagonists in the treatment of status epilepticus or perhaps pharmacoresistant epilepsy [19, 56]. However there are discrepant or even contradictory results with regard to the anticonvulsant effects in the used seizure models [20, 50]; see also [57]. On the other hand, direct anticonvulsant properties of P2X7R antagonists in traditional screening models or their potential usefulness in preventing epileptogenesis have not been examined in detail. To address this issue, we assessed possible anticonvulsant effects of a series of P2X7R antagonists: three CNS-permeable P2X7R antagonists; Brilliant Blue G (BBG) [58], JNJ-47965567 (JNJ) [59] and AFC-5128 (AFC) [60] as well as a natural compound-based derivative, tanshinone IIA sulfonate (TIIAS), as a negative allosteric modulator of the P2X7R [61]. Initially, we tested and compared the potency of the compounds using a fluorometric Ca^2+^ assay and different HEK293 cell lines stably expressing mouse, rat and human P2X7R. To identify anticonvulsant properties, the maximal electroshock seizure threshold test (MES-T) and the subcutaneous PTZ seizure threshold test (PTZ-T) in mice predictive for human generalized tonic-clonic seizures and generalized non-convulsive (myoclonic or absence) seizures, respectively, were chosen [62, 63]. Effects of co-administration with AEDs were also examined. A further objective was to investigate the influence of the four compounds on the PTZ-induced kindling development, a model of epileptogenesis [64, 65]. Anticonvulsant properties were also tested in fully kindled rats [64, 66]. In addition, we explored the effects of P2X7R antagonists on histopathological changes in the kindling model. Results here show synergistic acute anticonvulsant and antiepileptogenic effects of P2X7R antagonists, supporting consideration of this class of compound for epilepsy drug development [19]. Some of the results have previously been communicated in abstract form [67].

## Materials and Methods

### HEK293 Cell Culture

Human embryonic kidney 293 (HEK293) cells stably transfected with the human P2X7R (HEK_hP2X7_) were cultured in 75-cm^2^ cell culture flask to confluent monolayers with Dulbecco’s modified Eagle Medium (DMEM; c.c.pro, Oberdorla, Germany), containing 4.5 g/l d-glucose and supplemented with 10% foetal calf serum (FCS, Biochrom, Berlin, Germany), 2 mM L-glutamine (Sigma-Aldrich, Taufkirchen, Germany), and 50 μg/ml geneticin (Invitrogen, Karlsruhe, Germany). Cells were grown in a humidified incubator at 37°C in an atmosphere supplemented with 5% CO_2_. Mouse P2X7R cDNA was cloned from a C57BL/6 inbred strain into a pcDNA3.1/Zeo vector and the cloned cDNA was sequenced and stably expressed in HEK293 cells to obtain the HEK_mP2X7_ cell line. HEK293_mP2X7_ were cultured in DMEM under similar conditions as described above, but containing 100 μg/ml zeocin (Invitrogen). To obtain a stably transfected HEK_rP2X7_, the rat P2X7R was inducibly expressed in a HEK293 Flp-In T-REx cell line (Invitrogen), cultured in DMEM under similar conditions, but containing 15 μg/ml blasticidine and 100 μg/ml hygromycin B (both Invivogen, San Diego, CA, USA). Expression of the transgene was induced by supplementing the medium with 1 μg/ml tetracycline (Sigma-Aldrich, Taufkirchen, Germany) 24 h prior to the experiments.

### Fluorometric [Ca^2+^]_i_ Measurement

All fluorometric Ca^2+^ assays in cell suspensions were performed in 384-microwell plates (Corning, No. 3655, Lowell, MA, USA) with a fluorescence-imaging microplate reader (POLARstar Omega, BMG Labtech, Offenburg, Germany) (for details, see [61, 68]). Briefly, suspensions of HEK293 cells stably expressing human, mouse and rat P2X7R, respectively, were incubated with the fluorescent indicator fluo-4/AM (4 μM, Invitrogen, Darmstadt, Germany), in the dark for 30–45 min at 37°C, centrifuged (100xg for 3 min), and re-suspended in HEPES-buffered solution (HBS), containing 133 mM NaCl, 4.8 mM KCl, 1.2 mM KH_2_PO_4_, 1.3 mM CaCl_2_, 1 mM MgCl_2_, 10 mM HEPES and 10 mM d-glucose adjusted to pH 7.4 with NaOH, or in a similar solution without MgCl_2_ (as indicated).

To determine concentration-response relationships, test compounds were serially diluted down by a factor of two by using a programmable robotic liquid handling station (Freedom Evo 150, Tecan, Männedorf, Switzerland). The maximal final concentration was 50 μM (e.g., TIIAS), 5 μM (A43) and 0.5 μM (JNJ), respectively, depending on the compound and blocking potency at the corresponding kind of P2X7R. Microwell plates were repetitively scanned every 16 s in the fast scanning mode of the device. After 10 baseline cycles, ATP (in Mg^2+^-free HBS; final concentration of 1 mM) was injected into each well, and fluorescence intensities were followed after a delay of 2 min for up to 40 min (150 cycles) after ATP injection. Data were normalized to the baseline values before ATP application (F/F_0_).

### Animals (Seizure Tests)

All animal experiments were carried out in strict accordance with the recommendations in the Guide for the Care and Use of Laboratory Animals of the National Institutes of Health according to the European Communities Council Directive (86/609/EEC) and were approved by the Animal Welfare Offices (AbbVie Deutschland GmbH & Co KG) and the government of the State of Saxony (Ethics Committee, Landesdirektion Sachsen, Leipzig, Germany; Permit Number: TVV 26/13). The two screening seizure tests (MES-T and PTZ-T, see below) were carried out on male albino CD1 mice (breeding pairs supplied by the Medizinisch-Experimentelles Zentrum of the Medical Faculty, University of Leipzig, Germany), weighing 20 to 30 g. The experimental groups were chosen by means of a randomized schedule and each mouse was used for only one experiment. For the PTZ-kindling studies male Wistar rats were used (own breeding stock, Wistar/RjH: WI) weighing 190 to 220 g at the beginning of the experiments. The animals were kept in standard Plexiglas cages (10 mice and 3–4 rats per cage, respectively) with free access to commercial food pellets and drinking water at a constant temperature (22 ± 1°C), a relative humidity at 60% and a 12 h light-dark cycle with light on at 7.00 a.m. Mice and rats were daily inspected by the main researcher and personal from the animal house which included handling and body weight control. The specific criteria used to monitor animal health (e.g., inspection of fur and skin, eyes, breathing, excrements, movement disorders, weight loss, injuries, abscess, oedema etc.) are codified in a protocol with recommendations on possible actions. In addition, we had a protocol in place for the early euthanasia endpoints for animals which became severely ill during the experiments. The screening experiments were carried out between 8 and 12 h to avoid circadian influences. Mice were sacrificed by CO_2_ inhalation after termination of the seizure test, rats one day after the kindling procedure by CO_2_ inhalation and decapitation, respectively (see below).

### Maximal Electroshock Seizure Threshold Test (MES-T) in Mice

The stimulus train was applied via ear-clip electrodes (sinusoidal pulses 4–14 mA, 50 Hz, 0.2 s duration) by means of a constant current stimulator (rodent-shocker type 221; Hugo Sachs Elektronik, March-Hugstetten, Germany). The stimulus intensity was varied by an up-and-down method in which the current was increased or decreased in 1 nA-steps if the preceding animal did not or did show tonic hindlimb extension, respectively (for further details, see [69]). Groups of 20 mice were used. Control groups, which received the corresponding vehicle, were tested together with the compound-treated groups on the same experimental day. Current intensity-effect curves were obtained on the basis of the number of animals responding with the endpoint at the corresponding current value. The results are calculated as convulsive current fifty (CC_50_) values (current intensity in mA, necessary to produce tonic hindlimb extension in 50% of the mice tested) including the confidence limits for 95% probability.

### Pentylenetetrazol (PTZ) Seizure Threshold Test (PTZ-T) in Mice

Unrestrained mice, placed in separate full-view Plexiglas cages, were injected with the convulsant drug PTZ, a non-competitive GABA_A_ receptor antagonist [70], at a dose of 87 mg/kg s.c. in the neck and the appearance of the first generalized clonus (repeated clonic seizures of the fore- and hindlimbs lasting ≥3 s with an accompanying loss of righting reflex) was recorded during individual observation for 30 min (see [69]). The PTZ concentration was somewhat increased from normally 85 to 87 mg/kg s.c., because in a pilot experiment not all mice showed a generalized clonic seizure. Group sizes of 12 mice were used in all experiments. Control groups, which received vehicle, were tested together with the compound-treated group on the same experimental day. The number of animals in the group with a generalized clonic seizure and the latency time to its onset were analysed for statistical significance. In the absence of seizures within 30 min, the latency was taken as 1800 s.

### Pentylenetetrazol (PTZ)-Kindling (Rats)

For PTZ-kindling, the initial sub-convulsive dose of PTZ (35 mg/kg) was injected i.p. once every 48 h (on Monday, Wednesday, and Friday, three times a week). After vehicle or vehicle/compound pre-treatment, PTZ was injected and the rats were individually observed for 30 min. Behavioural seizure responses were scored according to a defined graduation from stage 0 to stage 5. Briefly, stage 0: no convulsive activity; stage 1: body twitching; stage 2: clonic forelimb convulsions without rearing; stage 3: severe bilateral forelimb clonus with full rearing; stage 4: generalized clonic seizures with loss of righting reflex; stage 5: generalized clonic-tonic seizures with loss of righting reflex and status epilepticus (≥ 2 min) (for the complete graduation extended with intermediate steps, see [64]). Rats received 20 successive PTZ-kindling stimulations. After an 8-day treatment-free interval, the animals were re-tested with PTZ (21^st^-25^th^ injection) and vehicle pre-treatment without the compounds. Results are presented as mean seizure stages within an experimental group (n = 9–12 rats). To avoid suffering and to prevent mortality associated with ongoing tonic-clonic seizures, rats received 5 mg/kg diazepam i.p. 2 min after seizure onset. Additional care was taken the hours following generalized tonic-clonic seizures and status epilepticus by providing the animals with soft food pellets and tap water if required. All efforts were made to minimize suffering.

### Studies on Fully Kindled Rats

Fully kindled rats (after having reached a series of at least 3 consecutive seizures of stage 3–5) were randomly allocated to groups of 6–12 animals and possible anticonvulsant effects of the compounds were investigated after acute administration. Results were expressed as mean seizure stages of a tested group in which the seizure behaviour after vehicle treatment (vehicle seizure stage) and after compound treatment (compound seizure stage), examined 3–4 days later, was matched. For comparison, the effectiveness of two antiepileptic drugs (carbamazepine and valproate) was also investigated.

### Plasma and Brain Concentration (Rats and Mice)

One day after kindling procedure, selected rats (groups with n ≥ 5) were again pre-treated with the compounds of interest and sacrificed at the assumed peak times of brain concentration by CO_2_ inhalation. Blood was collected intracardially by heart centesis via glass pipettes supplemented with heparin-Na (ratiopharm, Ulm, Germany; 10μl of 5.000 I.U./ml) and separated by centrifuging (1500xg for 10 min) at 6–8°C. The plasma was stored at -20°C in plastic tubes. After blood sample collection, rats were decapitated and brains were quickly dissected. After removing the adherent blood by tissue paper and cutting off the cerebellum, the samples were fresh frozen using liquid nitrogen. Brain tissue was stored at -80°C and delivered to the different laboratories on dry ice. For mice (groups with n = 6), a similar procedure was used for the preparation of plasma and brain samples. In experiments in which AFC was co-administered with carbamazepine or ethosuximide, drug concentrations in plasma and brain were estimated to detect potential pharmacokinetic interactions.

Samples from experiments with AFC were analysed at the Lead Discovery Center (Dortmund, Germany). Briefly, AFC, carbamazepine and ethosuximide were extracted from plasma and homogenized brain by protein precipitation using acetonitrile. Resulting filtrates were analysed by liquid chromatography tandem-mass spectrometry (LC-MS/MS) using a Prominence UFLC system (Shimadzu, Duisburg, Germany) coupled to a QTrap 5500 instrument (ABSciex, Darmstadt, Germany). The compounds were separated on a Zorbax Eclipse Plus C18 column (Agilent Technologies, Santa Clara, CA, USA) with an acetonitrile/water gradient containing 0.1% formic acid as solvent. Chromatographic conditions and mass spectrometer parameters were optimized for each analyte. Plasma and brain concentrations of the compounds were calculated by means of a standard curve.

Plasma and brain samples from experiments with JNJ-47965567 were quantified at the Neuroscience Therapeutic Area, Janssen Research & Development (San Diego, CA, USA) using an API 4000 MS/MS system (Applied Biosystems, Concord, ON, Canada) interfaced with an Agilent 1100 Series HPLC (Agilent Technologies). To measure the percentage of P2X7R brain occupancy of JNJ-47965567, *ex vivo* receptor binding autography was performed as recently described [71]. Briefly, 20 μm-thick hippocampal slices were prepared and P2X7R radioligand binding was determined at room temperature with 10 nM [^3^H] JNJ-54232334 (Janssen) in 50 mM Tris-HCl incubation buffer containing 0.1% bovine serum albumin. The non-specific binding was measured using adjacent sections incubated in the additional presence of 100 μM A-740003 (Tocris Bioscience, Bristol UK). Sections were incubated for 1 min, washed in ice-cold incubation buffer (4 times, 5 min each), followed by two dips in deionized water and rapidly dry under a stream of cold air before acquisition. Quantitative analysis was performed using the Tracer ß-Imager with M3 Vision software (Biospace Lab, Paris, France). *Ex vivo* receptor labelling was expressed as the percentage of receptor labelling in corresponding brain areas of vehicle-treated control animals.

### RNA Extraction and Quantitative Real-Time Polymerase Chain Reaction (RT-PCR)

RNA extraction was undertaken as previously described using Trizol (Invitrogen) [72]. Briefly, one microgram total RNA was used to generate cDNA by reverse transcription using Superscript II Reverse Transcriptase enzyme (Invitrogen). Quantitative RT-PCR was performed using a LightCycler 1.5 (Roche, Burgess Hill, West Sussex, UK) in combination with QuantiTech SYBR Green PCR kit (Qiagen, Crawley, West Sussex, UK) as per manufacturer's protocol and 1.25 μM of primer pair used. Data were analysed by LightCycler 1.5 software, data normalized to expression of β-actin and represented at relative quantification values. Primers were designed using Primer3 software (http://frodo.wi.mit.edu) and verified by BLAST (http://blast.ncbi.nlm.nih.gov/Blast.cgi). Primers sequences (Rattus norvegicus): β-Actin R: 5’- GGGGTGTTGAAGGTCTCAA, β-Actin F: 5’- TGTCACCAACTGGGACGATA; P2X7R R: 5’- AACCGCTTCTATCTGGAGCA, P2X7R F: 5’- GCTTGGGAAAAGTCTGCAAG.

### Western Blot Analysis

Western blotting was performed as previously described (see [50] and references therein). Whole hippocampi were homogenized in lysis buffer and protein concentration was measured. Protein samples (30 μg) were boiled in gel-loading buffer and separated on 10% sodium dodecyl sulfate polyacrylamide gel electrophoresis (SDS-PAGE) gels. Proteins were transferred onto polyvinylidene difluoride membranes (Bio-Rad, Hercules, CA, USA) and then incubated with antibodies against the following: anti-P2X7R antibody [1:200] (APR-004, Alomone, Jerusalem, Israel) and β-Actin [1:2000] (AB8227, Abcam, Cambridge, UK). Membranes were then incubated with horseradish peroxidase-conjugated secondary antibodies (Jackson, Suffolk, UK) and bands visualized using Supersignal West Pico Chemiluminescent Substrate (Pierce, Rockford, IL, USA). Images were captured using a Fuji-film LAS-300, densitometry performed using AlphaEaseFC4.0 software and data expressed as change relative to the loading control β-actin.

### (Immuno)histological Studies (Light Microscopy)

One day after the last kindling session (after the 25^th^ injection), vehicle/PTZ and compound/PTZ-treated rats as well as vehicle-only-treated or non-treated (naïve) rats as controls were transcardially perfused under deep CO_2_-anesthesia with 0.9% NaCl-solution, 2% paraformaldehyde (PFA, in 0.1 M sodium acetate buffer, pH 6.5) and 2% PFA/0.1% glutaraldehyde (in 0.1 M sodium borate buffer, pH 8.5) (see [73]). Brains were removed immediately and placed for 24 h in 2% PFA without glutaraldehyde (in 0.1 M sodium borate buffer, pH 8.5) at 4°C for post-fixation. Coronal sections (50 μm) were cut on a vibrating blade vibratome (Leica VT 1200 S, Wetzlar, Germany) and further processed for (immuno)histochemical staining.

To evaluate neuronal damage and cell loss, brain sections were mounted onto gelatine-coated slides and stained with 0.5% celestine blue/1% acidic fuchsin (both Sigma-Aldrich) according to standard procedures [73]. Damaged neurons can be visualized as dark red-violet stained cells without visible nuclei. Fluoro-Jade B staining was performed as described before [74]. Brain sections were incubated with 0.001% Fluoro-Jade B (Chemicon, Temezula, CA, USA) in 0.1% acetic acid for 30 min, washed with distilled water, air-dried, immersed in xylene and coverslipped using DePeX (Serva, Heidelberg, Germany). Sections were examined using a light microscope (Axioskop, Zeiss, Oberkochen, Germany) or a fluorescence microscope (Keyence BZ-9000, Neu-Isenburg, Germany).

For immunohistochemical studies, free-floating slices were incubated in 0.1 M Sørensen’s phosphate buffer (SPP) containing 1% H_2_O_2_ for 25 min. Then, after washing in blocking solution (SPP containing 0.3% Triton X-100, 2% normal horse serum or 2% normal goat serum) for 60 min at room temperature, slices were incubated with one of the primary antibodies, monoclonal mouse anti-GFAP [1:1.000] (Sigma-Aldrich), rabbit anti-Iba 1 [1:1.000] (Wako, Neuss, Germany) and rabbit anti-P2X7R [1:500] (APR-004, Alomone), respectively, in blocking solution overnight at 4°C in the dark (methodical procedure according [75]). After washing, the slices were incubated with the corresponding secondary antibodies, biotinylated horse anti-mouse immunoglobulin G (IgG) [1:65], and biotinylated goat anti-rabbit IgG [1:65], respectively (all from Vector, Burlingame, CA, USA) for 2 h at room temperature. After washing, incubation in elite ABC-Kit Vectastain^®^ [1:50] (Vector) for 1h at 4°C (all procedures in the dark) was followed by treatment with 3,3´-diaminobenzidine (DAB, Sigma-Aldrich, 0.07% in SPP, containing 0,001% H_2_O_2_), to visualize the labelled cells. The stained sections were processed through a series of graded ethanol and n-butylacetate and embedded with entellan (Merck). The immunoreactivity was evaluated using a light microscope (Axioskop, Zeiss) equipped with a CCD digital camera (AxioCam, ICc 1, Zeiss). For quantitative analysis of the GFAP and Iba 1-staining, images (20x magnification) from the hippocampal CA3 region of vehicle-only (controls) and vehicle/PTZ-treated animals (n = 10) were recorded at constant microscope adjustment and were evaluated with the LSM (LSM 510 Meta, Zeiss) using the semiautomatic image analysis software “Histogram” function. Significant differences were determined by calculating the abundance of densely stained pixels (pixel values lower than 67% of the background) using a two-tailed Wilcoxon rank sum test. Cell counts were also performed using the stored images (20x magnification).

### Immunofluorescence (Confocal LSM)

After washing in TBS and blocking (TBS containing 0.3% Triton X-100 and 5% FCS) for 60 min, free-floating slices were incubated with a mixture of three (or two, see below) primary antibodies, mouse anti-GFAP [1:1000] (Sigma-Aldrich), goat anti-Iba 1 [1:100] (Abcam) and rabbit anti-P2X7R [1:500] (APR-004, Alomone) in blocking solution for 48 h at 4°C. For simultaneous visualization of all three primary antibodies, slices were incubated with the corresponding (affinity-purified) antibodies, Alexa Fluor 647-conjugated donkey anti-mouse IgG [1:200], Cy2-conjugated donkey anti-goat IgG [1:200] and Cy3-conjugated donkey anti-rabbit [:800], all from Jackson) for 2 h at room temperature. At the end, slices were stained with Hoechst 33342 for 10 min (all in the dark) and further processed as described above. In some cases, double immunofluorescence staining was performed with rabbit anti-P2X7R and mouse anti-GFAP (see above), rabbit anti-P2X7R [1:500] and mouse anti-synaptophysin [1:200] (Sigma-Aldrich), or rabbit anti-P2X7R [1:500] and mouse anti-MAP2 [1:200] (Merck Millipore, Darmstadt, Germany) as described above, but using Cy2-conjugated donkey anti-mouse IgG [1:400] as secondary antibody both for GFAP and synaptophysin, or Cy5-conjugated goat anti-mouse [1:200] as secondary antibody for MAP2. Control experiments were performed without primary antibodies or by pre-adsorption of the antibodies with the immunizing peptides. The immunofluorescence was visualised by LSM at excitation wavelength of 488 nm (argon, green Cy2-fluorescence), 543 nm (helium/neon1, red Cy3-fluorescence), 633 nm (helium/neon2, Alexa Fluor 647-fluorescence, colour coded in blue) and 351–362 nm (argon-UV-laser, Hoechst 33342), respectively. Digital images were processed in Zeiss LSM Image Browser (Zeiss, version 2.80.1123) and Adobe Photoshop CS3 software (Adobe System, San Jose, CA, version 10.0) for adjusting the colour of each channel, scaling, resolution, contrast, and brightness, if necessary.

### Substances and Solutions (Materials)

The following pharmacological agents were used: adenosine 5’-triphosphate disodium salt (ATP), Brilliant Blue G (BBG), carbamazepine, ethosuximide (all from Sigma-Aldrich); A-438079 (3-[[5-(2,3-dichlorophenyl)-1*H*-tetrazol-1yl]methyl]pyridine hydrochloride hydrate), AZ-10606120 (*N*-[2-[[2-[(2-hydroxyethyl)amino]ethyl]amino]-5-quinolinyl]-2-tricyclo[3.3.1.13,7]dec-1-ylacetamide dihydrochloride), A-740003 (*N*-[1-[[(cyanoamino)(5-quinolinylamino)methylene]amino]-2,2-dimethylpropyl]-3,4-dimethoxybenzeneacetamide) (all from Tocris); AFC-5128 (indol-3-carboxamide derivative, chemical nomenclature disclosed; we thank Dr. M. Hamacher (Affectis Pharmaceuticals, Dortmund, Germany) for providing the compound); Ca-valproate dihydrate (AWD Dresden, ASTA Medica, Radebeul, Germany); diazepam (Rotexmedica, Trittau, Germany); JNJ-47965567 (*N*-[[4-(4-phenyl-piperazin-1-yl)tetrahydro-2*H*-pyran-4-yl]-methyl]-2-(phenyl-thio) nicotinamide) (Janssen); pentylenetetrazol (PTZ, Knoll, Ludwigshafen, Germany); tanshinone IIS sulfonic sodium (TIIAS, Wuxi Cima Science, Jiangsu, China).

For the fluorometric [Ca^2+^]_i_ measurement, stock solutions of drugs (10 mM) were prepared with deionized water (AZ-10606120) or DMSO (A-438079, AFC, BBG, JNJ, TIIAS). ATP-solution was freshly prepared in Mg^2+^-free HBS solution and adjusted to pH 7.4. Aliquots of stock solutions were stored at -20°C and freshly diluted with the appropriate HBS solution. The final DMSO concentrations never exceeded 0.5%, a concentration that had no discernible effect on ATP-induced increases in [Ca^2+^]_i_.

For the seizure tests (MES-T, PTZ-T, PTZ-kindling), BBG and TIIAS was dissolved in polyethylene glycol (PEG BioUltra, 400, Sigma-Aldrich), diluted to 20% with aqua (aqua ad iniectabilia, Braun, Melsungen, Germany). AFC was solubilized in ≥97% N,N-dimethylacetamide (DMA, Sigma-Aldrich), vortexed and diluted with 45% 2-hydroxypropyl-ß-cyclodextrin (ß-CD, Sigma-Aldrich) (final ratio of the vehicle 1:9). JNJ-47965567 was solubilized in two equivalents of 1N HCl, vortexed and sonicated (until the compound was mostly diluted) and then 30% of sulfobutylether-ß-cyclodextrin (SBE-ß-CD, Toronto Res. Chemicals, Toronto, Canada) was slowly added. The pH was adjusted with 1N NaOH (similar amount of NaOH as HCl), all with continuous stirring. PTZ was dissolved in 0.9% NaCl-solution, carbamazepine, ethosuximide and Ca-valproate were administered in a 2% (w/v) suspension of hydroxyethylcellulose. The drug solutions or suspensions were prepared immediately before use; all doses refer to the salts. Animals in the control group received equivalent volumes of the vehicle (in mice always 10 ml/kg, in rats 1 ml/kg [JNJ], 3 ml/kg [AFC] or 4 ml/kg [BBG, TIIAS], depending on the solubility of the different compounds). Doses and peak time effects were selected on the basis of effective doses from corresponding *in vivo* studies in the literature: BBG (e.g. [76, 77]), tanshinone IIA (e.g. [78, 79]) and from manufacturer’s experimental data: AFC, JNJ (see, [80]).

### Statistical Analysis

Data were analysed using SigmaPlot (vers. 12.0, Systat Software, Erkrath, Germany) and SPSS (IBM SPSS Statistics 20, Ehningen, Germany), respectively. Results are expressed as mean ± SEM. In the fluorometric Ca^2+^ assays, concentration-response curves were fitted by using a 4-parameter logistic function (Hill slope, E_min_, E_max_, IC_50_). The curves were approximated to a concentration value of 0.001 μM or lower (AZ-10606120 and JNJ) as the asymptotic 100% response. The IC_50_ value refers to 50% inhibition of the maximal [Ca^2+^]_i_ response (E_max_) induced by 1 mM ATP). In the MES-T, the current intensity inducing tonic hindlimb extensions in 50% of the mice (CC_50_ values with confidence limits for 95% probability) and the statistical significance was calculated using Probit analysis (SPSS). In the case of multiple comparisons between different groups α-correction was performed so that only differences with a *P* value of < 0.01 were considered significant. In the PTZ-T, the number of animals in the group with clonic seizures and the latency time were analysed using Fisher's exact probability test and the Mann-Whitney rank sum test, respectively. For multiple comparisons of the latency time a one-way ANOVA with the Holm-Sidak post hoc test was used. The influence of AFC on the plasma and brain concentrations of the antiepileptic drugs was statistical compared using the same procedure. In the PTZ-kindling model, the mean seizure stage within a group was calculated in relation to the number of PTZ-injections. Statistical comparison between vehicle- and compound-treated groups in this model was performed with two-way repeated measures ANOVA, followed by the Holm-Sidak post hoc test. The mean number of PTZ-injections needed to produce the first generalized seizure (≥ stage 3 convulsions) in the vehicle- versus compound-treated groups was statistically compared using a Mann-Whitney rank sum test. In fully kindled rats, the calculated mean seizure stage within a group after vehicle- and compound-treatment were compared using a paired *t*-test. *P* < 0.05 was the accepted minimum level of significance.

## Results

### Potency of the Four P2X7R Blocking Compounds to Inhibit P2X7R Response

**Fig 1:**
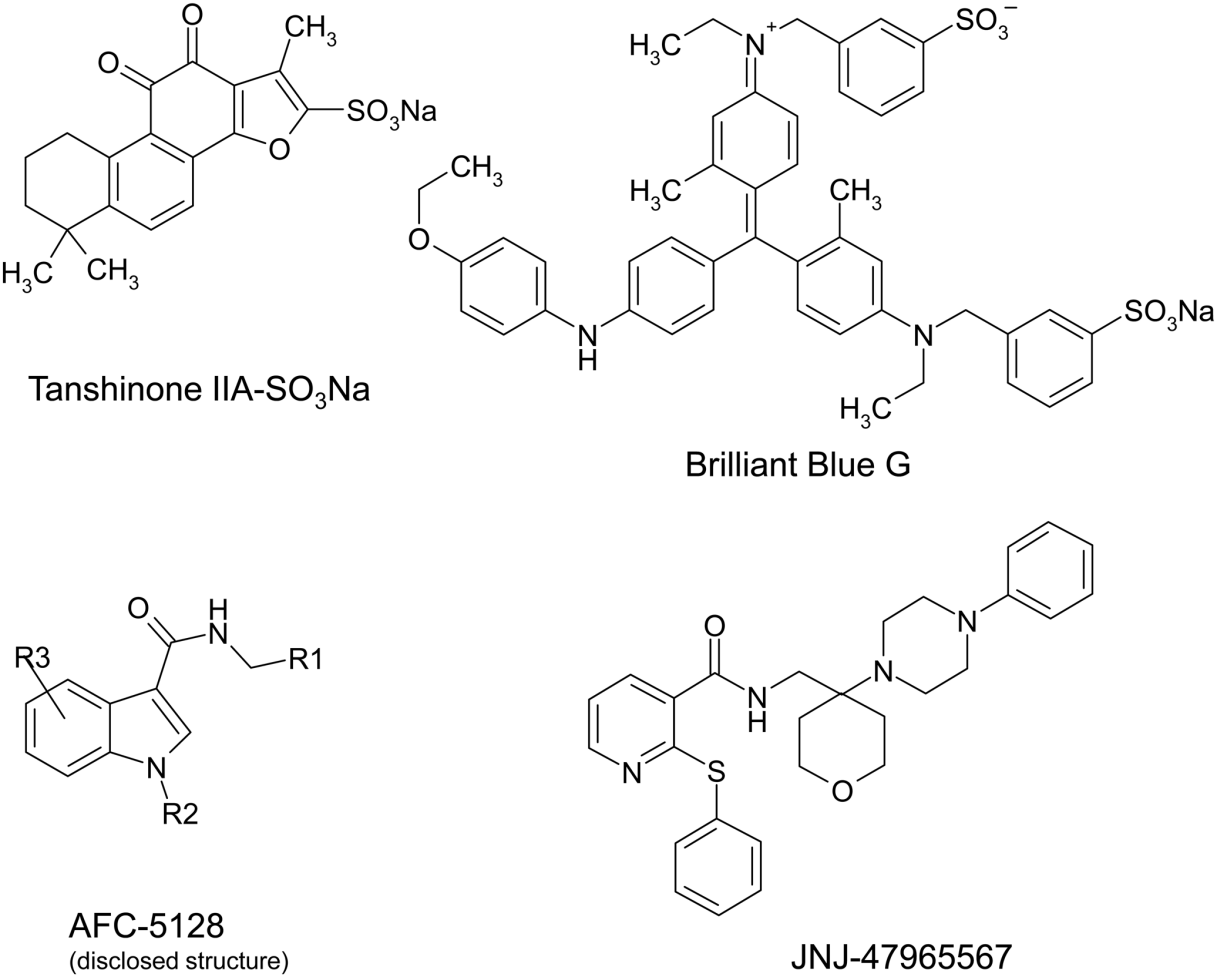
Chemical structure of the four investigated compounds.

**Fig 2:**
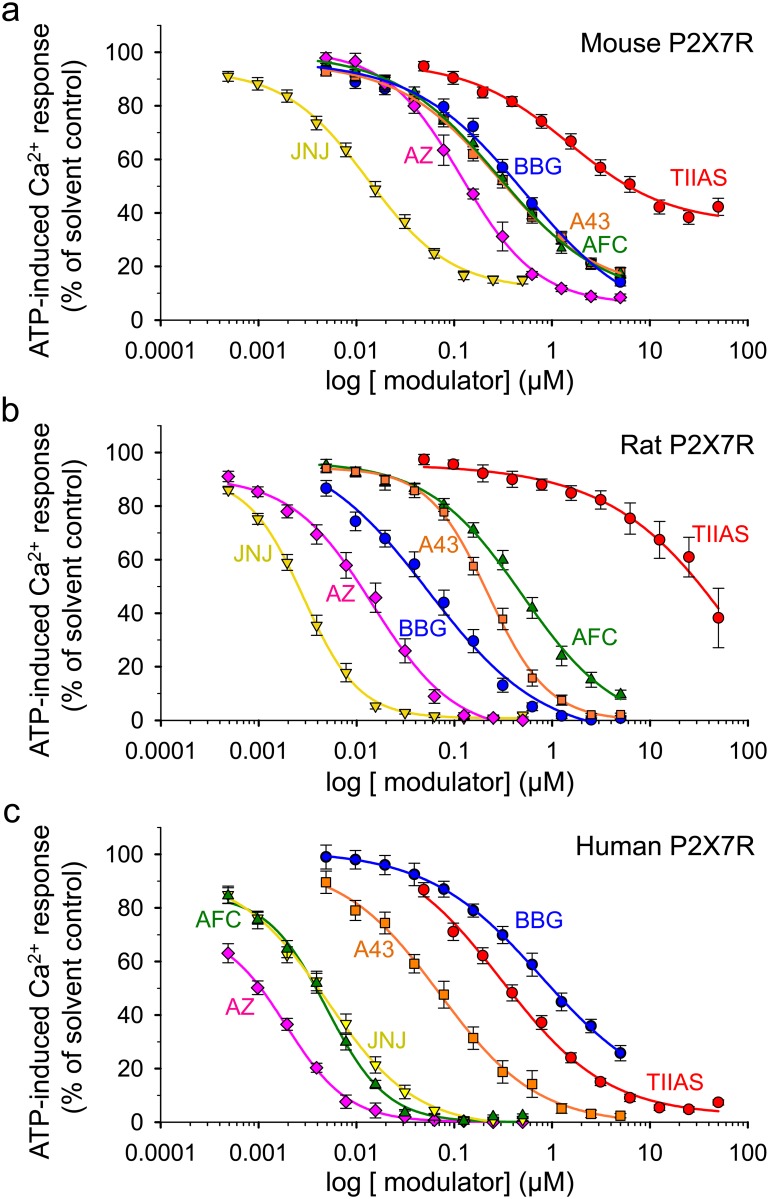
Concentration-dependent inhibition of ATP-induced [Ca^2+^]_i_ response. The studies were carried out in HEK293 cells, stably transfected with the mouse P2X7R (**a**), rat P2X7R (**b**), and human P2X7R (**c**), respectively (fluo-4 microfluorometry, cell suspension, microplate reader). Concentration-response curves are shown for the four tested compounds tanshinone IIA-SO_3_Na (TIIAS, red), Brilliant Blue G (BBG, blue), AFC-5128 (AFC, green), JNJ-47965567 (JNJ, yellow) as well as for the two P2X7R antagonists A-438079 (A43, orange) and AZ-10606120 (AZ, pink) for comparison. The curves were fitted by using a 4-parameter logistic function (SigmaPlot). Data points are expressed as mean ± SEM (n = 5–7 independent experiments performed as duplicates). ATP-induced Ca^2+^ responses are calculated in percentage of the respective controls with solvent alone.

The following studies were carried out with the four selected P2X7R blocking compounds TIIAS, a water-soluble derivative of the natural compound tanshinone found in the roots of red sage, the classical blocker BBG as well as AFC and JNJ, two novel potent P2X7R-specific antagonists. The chemical structure of the compounds is shown in Fig 1. The first experiments were conducted to evaluate the potency of these compounds in comparison with two established P2X7R antagonists, A-438079 and AZ-10606120, using a fluorometric Ca^2+^ assay and various HEK293 cell lines stably expressing mouse (HEK_mP2X7_), rat (HEK_rP2X7_) or human (HEK_hP2X7_) P2X7R, respectively. In the transfected HEK293 cells, loaded with the Ca^2+^ indicator dye fluo-4, high ATP concentrations (1 mM) induced a long-lasting Ca^2+^ entry recorded as an increase in fluorescence intensity during the recording period. Fig 2 shows the concentration-dependent blocking effects (concentration-response curves) of TIIAS, BBG, AFC and JNJ, which may be different in HEK_mP2X7_ (2a), HEK_rP2X7_ (2b) and HEK_hP2X7_ (2c) cells, respectively. (For illustration, representative recordings of Ca^2+^ entry traces from single 384-well plate measurements are added in S1 Fig). The potent inhibition by AZ-10606120 and A-438079 confirms that P2X7R accounts for the ATP-triggered Ca^2+^ entry. The potency (half-maximal inhibitory concentration IC_50_ ± SEM) reveals remarkable species differences of the compounds tested (Table 1). The natural compound derivative TIIAS shows potent blocking effects on human P2X7R (IC_50_ = 0.3 μM), but only poor effects on mouse and rat P2X7R. BBG is known to be more selective for rat P2X7R (IC_50_ = 50 nM) than for mouse or human P2X7R. The novel compound AFC shows high potency on the human P2X7R (IC_50_ = 5 nM) and moderate on mouse (IC_50_ = 280 nM) and rat P2X7R (IC_50_ = 500 nM). The potency of JNJ is high on rat (IC_50_ = 3 nM), human (IC_50_ = 5 nM) as well as on mouse P2X7R (IC_50_ = 10 nM).

**Table 1:** Potency (IC_50_ ± SEM in μM) of the four investigated compounds in comparison with A-438079 and AZ-10606120 (fluorometric Ca^2+^ assay).

Substance	Mouse P2X7R	Rat P2X7R	Human P2X7R
Tanshinone TIIAS	≥ 6	≥ 30	0.31 ± 0.08
Brilliant Blue G	0.53 ± 0.15	0.05 ± 0.01	0.98 ± 0.07
AFC-5128	0.28 ± 0.03	0.5 ± 0.08	0.005 ± 0.0004
JNJ-47965567	0.01 ± 0.001	0.003 ± 0.0001	0.005 ± 0.0005
A-438079	0.28 ± 0.02	0.22 ± 0.01	0.07 ± 0.009
AZ-10606120	0.12 ± 0.005	0.02 ± 0.002	0.001 ± 0.0001

Studies were carried out with three different HEK293 cell lines stably expressing mouse (HEK_mP2X7_), rat (HEK_rP2X7_) or human (HEK_hP2X7_) P2X7R, respectively (fluo-4 microfluorometry, cell suspension, microplate reader).

### Effects of P2X7R Antagonists on the Threshold for Electroshock-Induced Tonic Seizures

**Fig 3:**
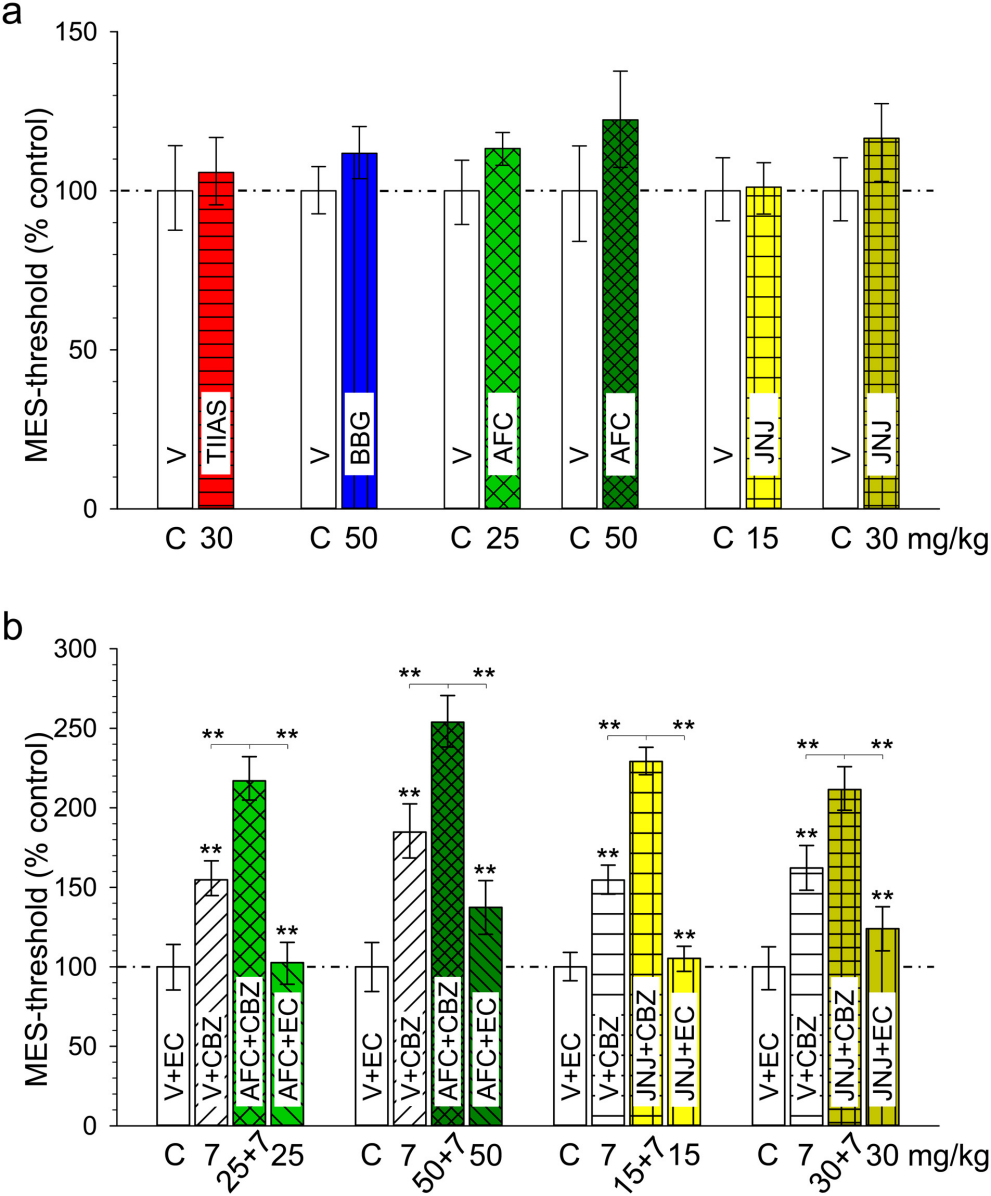
Maximal Electroshock Seizure threshold test (MES-T) in mice. **(a)** Effects of tanshinone IIA SO_3_Na (TIIAS), Brilliant Blue G (BBG), AFC-5128 (AFC) and JNJ-47965567 (JNJ) on the threshold for tonic (hindlimb extension) electroshock seizures. The columns represent the CC_50_ values with confidence limits for 95% probability of compounds (20 animals per group), expressed in percent of the parallel estimated control thresholds (C = 100%, open columns). Controls received the corresponding vehicle i.p.; doses of compounds (mg/kg i.p.) are indicated below the columns. TIIAS and BBG were administered 45 min before and AFC and JNJ 30 min before threshold determination. Mean of control thresholds: 4.6 to 5.4 mA. There is not a statistically significant difference (Probit analysis). **(b)** Influence of AFC-5128 (AFC) and JNJ-47965567 (JNJ) on the anticonvulsant efficacy of carbamazepine (CBZ) in the MES-T (representation as in (a), CC_50_ values and confidence limits, expressed in percent of the parallel estimated control thresholds (C = 100%, open columns). AFC (25 or 50 mg/kg) and JNJ (15 or 30 mg/kg) were administered s.c. 30 min before threshold determination (additional injection of ethylcellulose [EC] i.p. 30 min instead of CBZ 7 mg/kg); the antiepileptic drug was given i.p. 30 min before (injection of vehicle s.c. 30 min instead of AFC or JNJ). Doses of compounds are indicated below the columns. Mean of control thresholds (injection of vehicle s.c. and ethylcellulose i.p., 30 min before each): 5.34 (AFC 25 mg/kg), 4.89 (AFC 50 mg/kg), 5.44 (JNJ 15 mg/kg), and 4.52 mA (JNJ 30 mg/kg), respectively. Significance level: **P < 0.01 (Probit analysis).

Possible anticonvulsant effects were investigated using the MES-T and the PTZ-T (see next paragraph) in mice. These models are regarded to be predictive for human generalized tonic-clonic seizures and generalized non-convulsive (absence or myoclonic) seizures, respectively [62, 63]. In the MES-T, the threshold for tonic (hindlimb extension) electroshock seizures was not markedly influenced by the four tested P2X7R blocker at the assumed peak time (Fig 3a). There was, however, a tendency for a small, but non-significant increase in seizure threshold at the higher concentrations of AFC (50 mg/kg) and JNJ (30 mg/kg); in the case of AFC mild sedative effects were observed. On the other hand, the clinically used anticonvulsant drug, carbamazepine (7 mg/kg i.p.) administered alone, caused a significant increase of the electroconvulsive threshold by about 50%. As shown in Fig 3b, co-administration of AFC (25 and 50 mg/kg s.c.) as well as JNJ (15 and 30 mg/kg s.c.) with carbamazepine significantly increased the anticonvulsant effectiveness as compared to carbamazepine alone.

### Effects of P2X7R Antagonists on PTZ-Induced Generalized Clonic Seizures (PTZ-T)

In the PTZ-T test in mice, all four compounds showed no significant protection from PTZ-induced generalized clonic seizures. However, BBG, TIIAS and JNJ (AFC) at higher doses tended to increase the latency to the onset of the first clonic seizures (Table 2). The incidence of death was 0–2 mice per treatment group within the 30 min observation period.

**Table 2:** Pentylenetetrazol seizure threshold test (PTZ-T) in mice.

			Clonic seizures:	
Vehicle and Substance	Dose (mg/kg)	# of mice/ with seizure	Latency to the first gen. clonus (min) (% of control)	Cases of death (within 30 min)
Vehicle	-	12/12	5.00 ± 1.04	0
TIIAS	30	12/11	10.08 ± 2.64 (201.6 ± 52.8%)	0
Vehicle	-	12/11	6.77 ± 2.23	0
BBG	50	12/10	13.91 ± 2.38* (205.5 ± 35.2%)	2
Vehicle	-	12/10	18.67 ± 3.03	1
AFC-5128	25	12/9	21.97 ± 2.81 (117.7 ± 15.1%)	0
Vehicle	-	12/11	13.99 ± 2.81	1
AFC-5128	50	12/8	20.60 ± 2.60 147.2 ± 18.6%)	0
Vehicle	-	12/12	6.94 ± 1.31	1
JNJ-47965567	15	12/12	7.31 ± 2.19 (105.3 ± 31.6%)	2
Vehicle	-	12/12	7.13 ± 1.49	1
JNJ-47965567	30	12/10	13.66 ± 2.94 (191.6 ± 41.2%)	1

Influence of tanshinone IIA-SO_3_Na (TIIAS), Brilliant Blue G (BBG), AFC-5128 and JNJ-47965567 on PTZ-induced clonic seizures. Vehicle and substance combinations were applied i.p. 30 min (or 45 min in combinations with TIIAS and BBG) before PTZ injection (87 mg/kg s.c.). PTZ seizure latency is given as mean ± SEM. No statistical significance between the number of mice with/without seizures (Fisher’s exact probability test); Seizure latency: *P < 0.05 (Mann-Whitney rank sum test).

In this test, the effective AED ethosuximide alone (at the lower dose of 100 mg/kg i.p.) protected some animals against generalized clonic seizures and significantly increased the latency to the first clonic seizures (Table 3). The co-administration of ethosuximide with AFC and JNJ, respectively, did not produce stronger protective effects against generalized clonic seizures, however a further increase of the latency to generalized clonic seizures compared to the vehicle-treated controls was observed. The increase of the latency as compared to ethosuximide alone did not reach statistical significance.

**Table 3:** Influence of AFC-5128 as well as JNJ-47965567 upon the protective action of ethosuximide against PTZ-induced clonic seizures (PTZ-T) in mice.

			Clonic seizures:	
Vehicle and Substance	Dose (mg/kg) (i.p.)	# of mice/ with seizures	Latency to the first gen. Clonus (min) (% control)	Cases of death (within 30 min)
Ethylcellulose +Vehicle	- -	12/12	4.53 ± 0.56 (100.0 ± 12.4%)	3
Ethylcellulose +AFC-5128	- 25	12/10	8.06 ± 1.48 (177.9 ± 32.7%)	2
Ethosuximide +Vehicle	100 -	12/8^+^	14.16 ± 2.98* (312.6 ± 65.8%)	1
Ethosuximide +AFC-5128	100 25	12/7^+^	18.84 ± 3.01*** (415.9 ± 66.4%)	0
Ethylcellulose +Vehicle	- -	12/11	10.83 ± 2.67 (100.0 ± 24.7%)	2
Ethylcellulose +AFC-5128	- 50	12/10	14.31 ± 2.88 (132.1 ± 26.6%)	1
Ethosuximide +Vehicle	100 -	12/7^+^	21.72 ± 3.03* (200.6 ± 27.0%)	1
Ethosuximide +AFC-5128	100 50	12/7^+^	26.60 ± 2.29*** (245.5 ± 21.1%)	0
Ethylcellulose +Vehicle	- -	12/12	4.74 ± 0.64 (100.0 ± 13.5%)	2
Ethylcellulose +JNJ-47965567	- 15	12/12	5.19 ± 1.19 (109.4 ± 25.1%)	3
Ethosuximide +Vehicle	100 -	12/8^+^	11.58 ± 3.57* (244.3 ± 75.3%)	0
Ethosuximide +JNJ-47965567	100 15	12/11	9.76 ± 2.82* (205.9 ± 59.4%)	0
Ethylcellulose +Vehicle	- -	12/12	6.14 ± 1.34 (100.0 ± 21.8%)	2
Ethylcellulose +JNJ-47965567	- 30	12/10	9.42 ± 2.89 (153.4 ± 47.1%)	1
Ethosuximide +Vehicle	100 -	12/8^+^	12.83 ± 3.14* (209.0 ± 51.1%)	1
Ethosuximide +JNJ-47965567	100 30	12/6^+^	19.85 ± 3.64** (323.3 ± 59.3%)	1

Ethylcellulose, vehicle and substance combinations were all applied i.p., 30 min before PTZ injection (87 mg/kg s.c.). PTZ seizure latency is given as mean ± SEM. Statistical significance between control and drug data: ^+^P<0.05 (Fisher’s exact probability test); *P < 0.05, **P < 0.01, ***P < 0.001 (one-way Anova, Holm-Sidak post hoc test).

### Influence of P2X7R Antagonists on the Development of PTZ-Induced Kindling

PTZ-induced kindling in rats is a known animal model of epileptogenesis [64, 65]. Repeated injections of an initial subconvulsant dose of the chemoconvulsant PTZ (35 mg/kg i.p., once every 48h) induces a progressive development of behavioural seizure phenomena beginning with the second to third administration. After 7 to 9 PTZ-injections, 60–80% of the animals had generalized seizures (mean seizure stage ≥ 3 [bilateral forelimb clonic convulsions] or more). It should be noted, however, that the individual animal responses are variable and the increase of seizure severity in the group over the course of kindling is not directly linear. After an 8-day treatment-free interval, the kindling phenomenon not only persisted, but most animals showed stronger score responses at the first post-interval PTZ-injection (21^st^ PTZ-injection in all).

**Fig 4:**
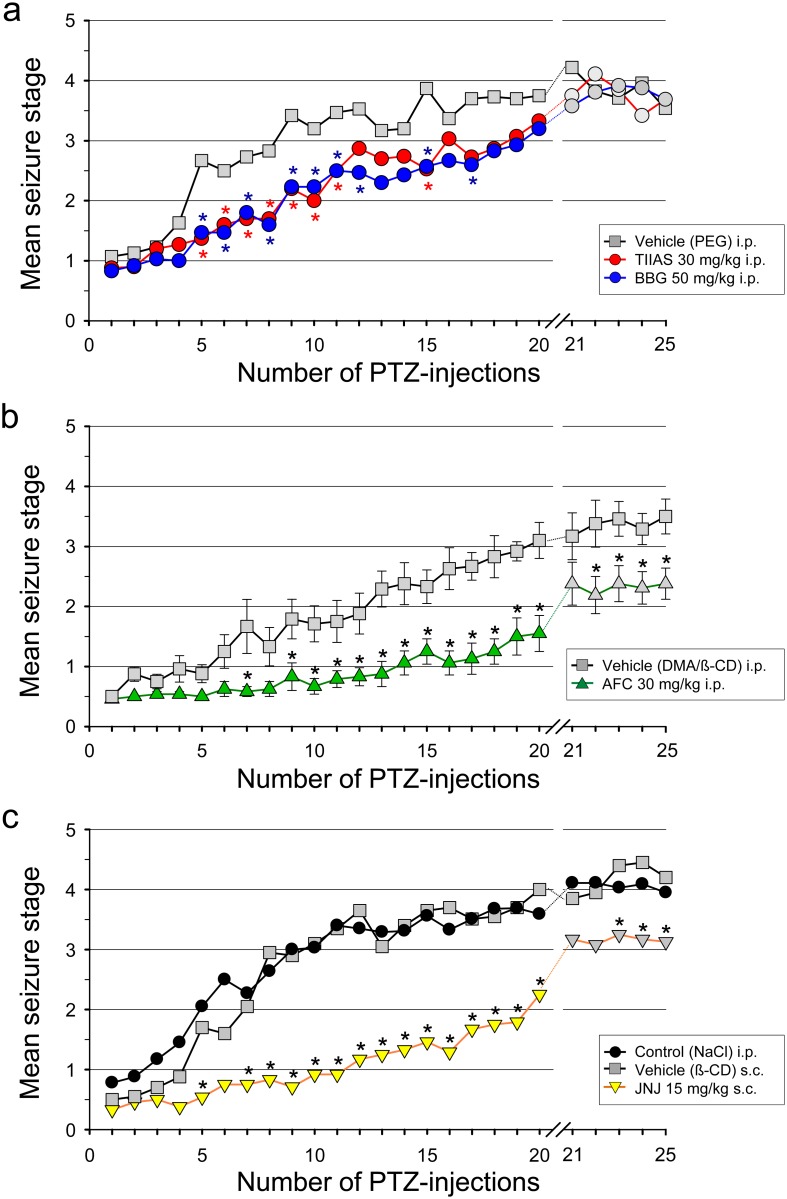
PTZ-induced kindling in rats. Influence of the four investigated compounds on the development of PTZ-kindling. **(a)** *First series*: Tanshinone IIA SO_3_Na (TIIAS, 30 mg/kg) and Brilliant Blue G (BBG, 50 mg/kg) were administered i.p. 45 min before PTZ with vehicle (20% PEG 400). **(b)** *Second series*: AFC-5128 (AFC, 30 mg/kg) was given i.p. 45 min before PTZ with vehicle (DMA/β-CD). **(c)** *Third series*: JNJ-47965567 (JNJ, 15 mg/kg) was given s.c. 30 min before PTZ with vehicle (SBE-β-CD). The control groups (n = 12) received the corresponding vehicle alone. In addition, in the third series a further control group pre-treated with 0.9% NaCl-solution was included. PTZ (35 mg/kg i.p.) was injected once every 48 h (three times a week on Monday, Wednesday and Friday) for 20 successive sessions. After 20 PTZ-injections and an 8-day interruption of the kindling procedure, the rats were further kindled with only vehicle pre-treatment instead of the compounds (21^st^ to 25^th^ PTZ-injection). Values shown represent mean seizure stages. In order to keep the curves clear from the others, error bars (± SEM) were indicated only in Fig 4b. (SEM in all line graphs was not higher than ± 0.4.) Significance level: *P <0.05 (two-way repeated measures ANOVA, Holm-Sidak post hoc test).

In the first series of investigations, BBG and tanshinone exhibited a moderate, yet significant retarding effect on kindling development, visibly from the 5^th^ to the 11^th^ PTZ-injection, in comparison to the vehicle (PEG 400)-treated control (Fig 4a). However, this effect diminished later and was found to be transient. After an 8-day treatment-free interval, no differences of the mean seizure stages between the formerly compound- and the vehicle-treated groups can be observed. In the second series, AFC (30 mg/kg i.p.) significantly reduced the progression of seizure development to higher seizure stages visible from the 7^th^ or rather 9^th^ to the 20^th^ PTZ-injection and showed a remarkable long-lasting effect after the 8-day treatment-free interval (Fig 4b). However, the stage of increased susceptibility induced by the development of PTZ- kindling persisted. The proposed vehicle mixture (dimethylacetamid/hydroxypropyl-ß-cyclodextrin), however, seems also to delay slightly the seizure development. Similarly, in the third series, JNJ (15 mg/kg s.c.) significantly reduced the PTZ-kindling development (Fig 4c). In the last series, an additional control group was tested with injections of NaCl-solution instead of vehicle, showing a similar increase of mean seizure stages during the kindling procedure. Moreover, the curve progression in the NaCl-treated control group is well comparable with the findings documented previously of our group with “WIST/Prob” Wistar rats (see [64]).

**Fig 5:**
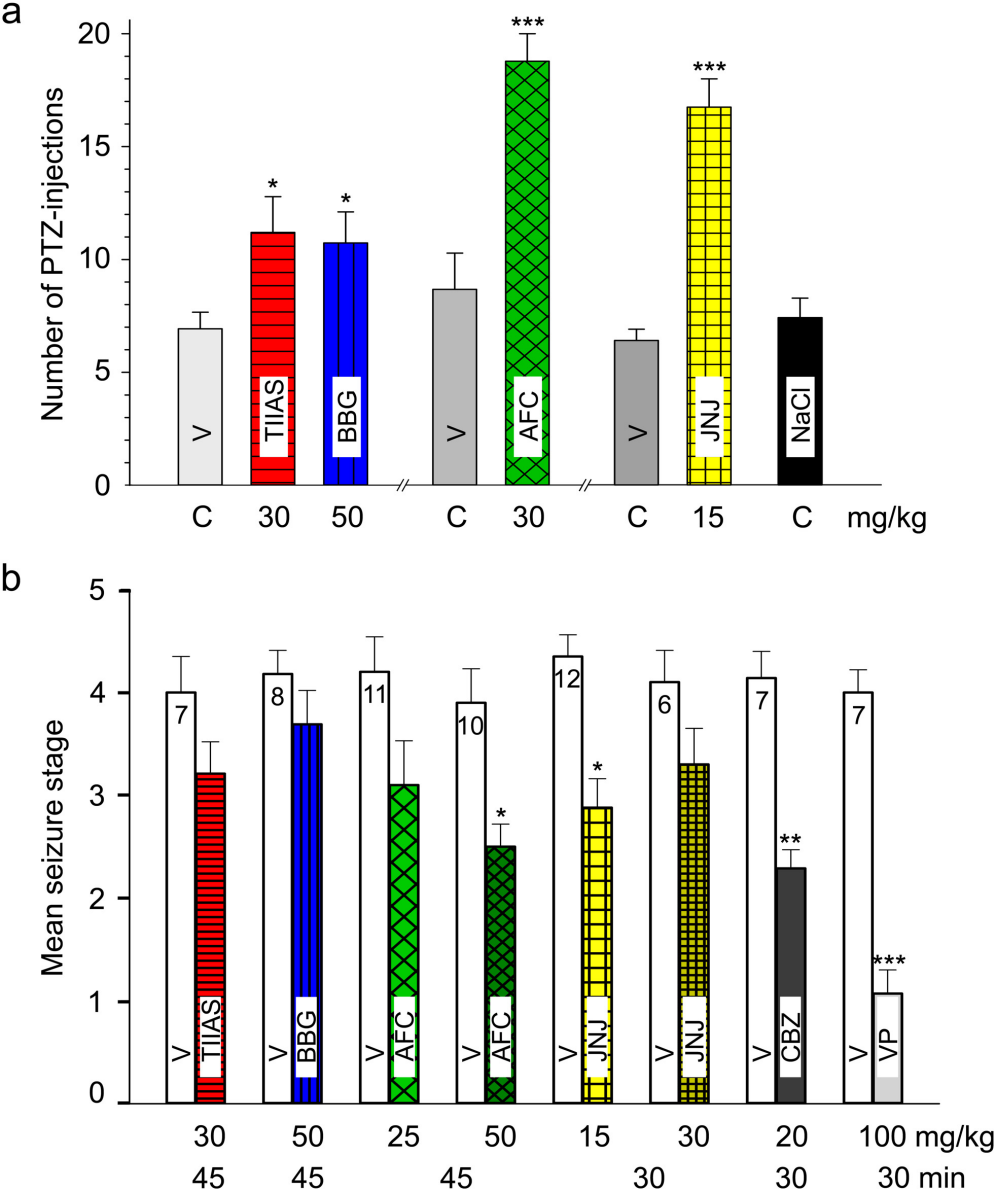
PTZ-induced kindling in rats. **(a)** Mean number of PTZ-injections needed to produce the first generalized seizure in the three series of PTZ-kindling (see Fig 4). *Left part*: impact of Tanshinone IIA SO_3_Na (TIIAS) and Brilliant Blue G (BBG); *Middle*: impact of AFC-5128 (AFC); *Right part*: impact of JNJ-47965567 (JNJ) in comparison with the corresponding vehicle controls (n = 12). Animals, which showed no generalized seizure (< stage 3) up to the 20^th^ PTZ-injection, were calculated with “20”. Significance level: *P <0.05, ***P < 0.001 (Mann-Whitney rank sum test). **(b)** Effects of tanshinone IIA-SO_3_Na (TIIAS), Brilliant Blue G (BBG), AFC-5128 (AFC) and JNJ-47965567 (JNJ) on PTZ-induced seizures in fully PTZ-kindled rats in comparison with carbamazepine (CBZ) and Ca-valproate (VP). Each pair of columns represents the mean seizure stage (+ SEM) of a tested group, after vehicle treatment (vehicle seizure stage) and after compound treatment (compound seizure stage), indicated as white and coloured column, respectively). The number of animals within the group is shown on the top of the columns, the doses of compounds and the time of pre-medication before PTZ-injection are shown below the columns. Significance level: *P < 0.05, **P < 0.01, ***P < 0.001 (paired *t*-test).

The mean number of PTZ-injections needed to produce generalized seizures (≥ stage 3 convulsions) in the three PTZ-kindling series is summarized in Fig 5a. The groups pre-treated with TIIAS, BBG, AFC or JNJ required significantly more PTZ-injections to reach the first generalized clonus than the vehicle-only control groups pre-treated with the corresponding vehicle.

### Influence of P2X7R Antagonists on Seizures in Fully Kindled Rats

In an additional set of experiments, the four selected P2X7R blocker were also tested for effects on acute PTZ-induced seizures in fully kindled rats as a model for primary generalized tonic-clonic seizures [64, 66]. PTZ-kindled animals, taken from the vehicle/PTZ-treated control groups at the end, were selected when they had reached at least three consecutive seizures of stage 3 to 5. In general, a group of rats (n ≥ 6) was tested with the corresponding vehicle (vehicle seizure stage; white columns) and 3–4 days later with the selected compound (compound seizure stage, coloured columns) (Fig 5b). TIIAS (30 mg/kg i.p.), BBG (50 mg/kg i.p.), AFC (25 mg/kg i.p.) and the higher dose of JNJ (30 mg/kg s.c.) revealed a tendency to reduce the mean seizure stage, whereas AFC (50 mg/kg i.p.), pre-drug to post-drug values: 3.9 to 2.5 and the lower concentration of JNJ (15 mg/kg s.c.): 4.4 to 2.9 reduced the mean seizure stage significantly. In addition, the efficacy of the two AEDs, carbamazepine (20 mg/kg i.p.): 4.1 to 2.3 and Ca-valproate (100 mg/kg i.p.): 4.0 to 1.1 was also examined.

### Plasma and Brain Levels of P2X7R Antagonists as well as Effects of AFC on the Levels of Carbamazepine and Ethosuximide

The mean blood plasma and brain concentrations of the tested compounds determined after the end of the PTZ-kindling procedure in rats were for TIIAS (30 mg/kg i.p., 45 min before) 31.1 ± 5.9 μM and 0.5 ± 0.1 μM (n = 5), for BBG (50 mg/kg i.p., 45 min before) 1.6 ± 0.3 and 0.02 ± 0.002 μM (n = 7), and for AFC (30 mg/kg i.p., 45 min before) 19.4 ± 3.7 μM and 15.5 ± 4.0 μM (n = 8), respectively. These data give values for the brain to plasma ratio of 0.016, 0.013 and 0.80, respectively. On the other hand, the mean blood plasma and brain concentrations in rats for JNJ (15 mg/kg s.c., 30 min before) were 4.0 ± 1.0 μM and 5.0 ± 1.1 μM (n = 5), respectively, the brain to plasma ratio was 1.2. Furthermore, the P2X7R brain occupancy of JNJ determined by P2X7R radioligand binding autoradiography was 98.0 ± 2.0% at this dose. The measured brain concentrations of the two potent antagonists AFC and JNJ are high enough for blocking the P2X7R *in vivo*. The CNS penetration of TIIAS and BBG seem to be much smaller and reached only the nM-range. Thus, they may only partly block the P2X7R when given systematically.

In the case of co-application of AFC with CBZ (MES-T, mice), the plasma concentrations of the AED were not significantly modulated, but the brain concentrations were significantly lowered in comparison to the values of CBZ alone (from 22.8 ± 1.5 μM to 16.2 ± 0.6 or 17.2 ± 1.7 μM with AFC 25 or 50 mg/kg s.c., respectively (n = 6 each; one-way ANOVA). These findings suggest that pharmacokinetic interactions between AFC and CBZ may occur, however, the measured decrease of the CBZ brain concentrations by both doses of AFC cannot give an explanation for the marked increase of the tonic seizure threshold in the MES-T (see Fig 3b). In addition, the co-determined blood and brain concentrations of AFC were 13.4 ± 1.1 and 7.8 ± 0.7 μM (25 mg/kg s.c., 30 min before) as well as 21.9 ± 1.9 and 13.5 ± 2.3 μM (50 mg/kg s.c., 30 min before, n = 6 each). On the other hand, the co-application of AFC with ethosuximide (PTZ-T, mice) did not significantly modulate the blood concentrations of the AED, but the higher dose of AFC (50 mg/kg i.p.) significantly increased the brain concentrations of ethosuximide (from 377.3 ± 14.3 μM to 453.9 ± 18.7 μM, n = 6 each; one-way ANOVA). The protective action of ethosuximide, however, was not markedly increased by the co-application in the PTZ-T test (see Tab. 3a). In addition, the co-determined blood and brain concentrations of AFC were 22.4 ± 1.2 and 19.9 ± 2.4 μM (25 mg/kg i.p., 30 min before) as well as 57.3 ± 6.5 and 42.7 ± 3.8 μM (50 mg/kg i.p., 30 min before, n = 7 each), about two orders of magnitude higher than the estimated IC_50_ value in HEK_mP2X7_ cells.

### Effect of PTZ-Kindling on P2X7R Messenger RNA and Protein Level

Next, we investigated whether the generalized tonic-clonic convulsions in the PTZ-kindling model are associated with up-regulation of the P2X7R. The hippocampus was removed from control and kindled rats and P2X7R expression analysed by RT-PCR and Western blot analysis. Quantitative RT-PCR revealed no clear differences in *P2rx7* messenger RNA levels 24h after the 10^th^ and 25^th^ PTZ injection in comparison to naïve and saline-treated control rats (S2a Fig). Western blot analysis showed no increase in P2X7R levels in the hippocampus after the 10^th^ injection of PTZ and only a marginal, but non-significant increase of P2X7R protein after the 25^th^ PTZ injection between the saline-treated and the PTZ-treated groups, respectively (S2b Fig).

### Histology and Immunohistochemical Studies

**Fig 6:**
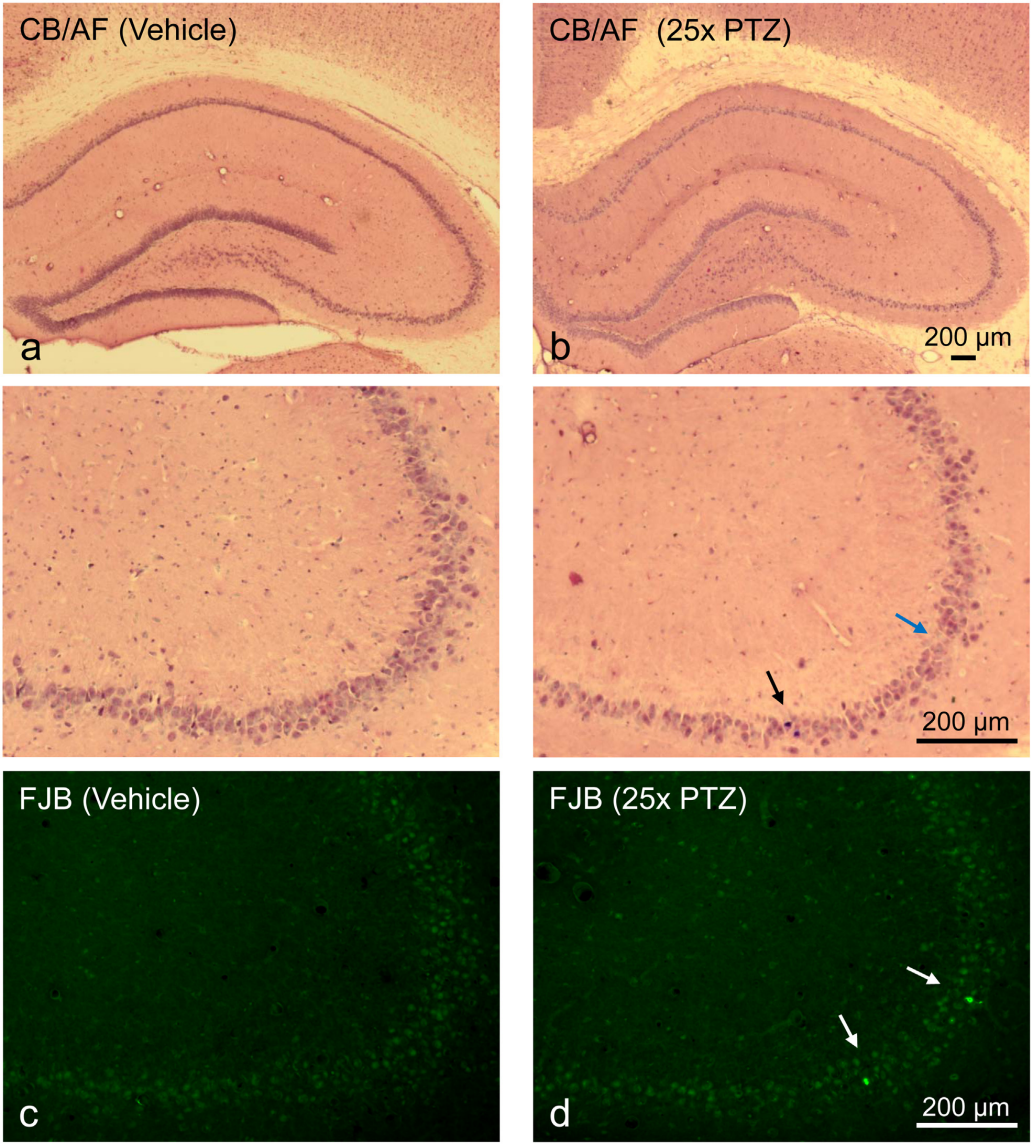
Representative photomicrographs of celestine blue/acid fuchsin (CB/AF) and Fluoro-Jade B (FJB)-stained coronal sections from the right dorsal anterior hippocampus of rats. **(a)** CB/AF staining, control animal, 24h after the 25^th^ vehicle injection (20% PEG 400, vehicle-only group), higher magnification view of CA3 pyramidal cell layer below. **(b)** CB/AF staining, PTZ-treated rat, 24h after the 25^th^ PTZ injections (vehicle/PTZ group), higher magnification view below. Damaged cells (dark red-violet, black arrow) are rarely observed, however, in some cases cell-free gaps (blue arrow) can be seen indicating cell loss (see magnification of CA3). **(c)** FJB staining, control animal. **(d)** FJB staining, PTZ-treated rat. Bright green-fluorescent FJB-positive cells (white arrows) are rarely observed.

There was evidence of minor neuronal damage in the dorsal hippocampus of vehicle/PTZ-treated rats at the end of the PTZ-kindling studies, as identified by celestine blue/acidic fuchsin (Fig 6a and 6b) or Fluoro-Jade B staining (Fig 6c and 6d).

**Fig 7:**
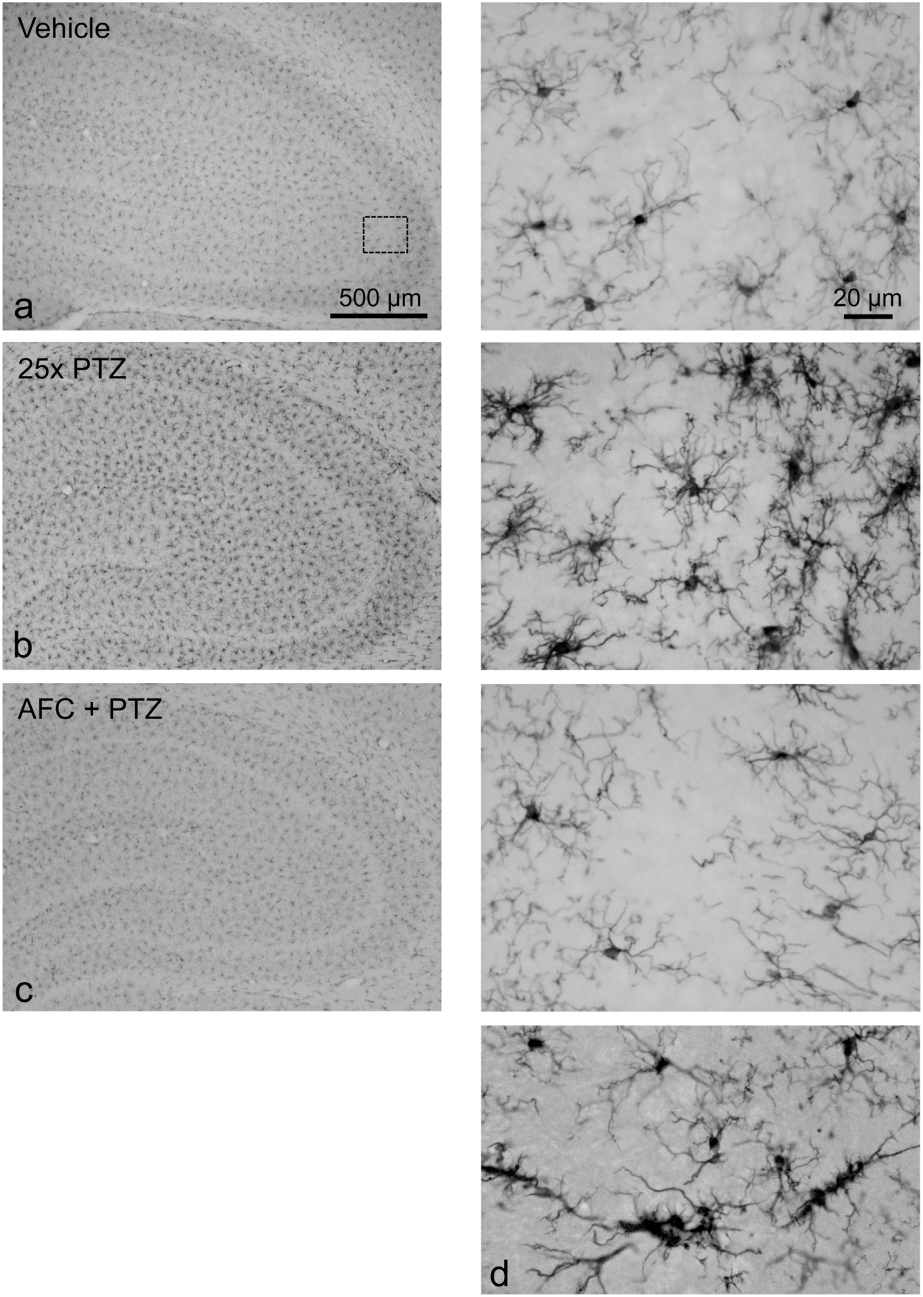
Representative light microscopy photomicrographs showing Iba 1-immunopositive microglial cells from the right dorsal anterior hippocampus of the rat. (**a**) Control animal, 24h after the 25^th^ vehicle injection (20% PEG 400, vehicle-only group); higher magnification view of CA3 subfield (outlined area) on the right. (**b**) PTZ-kindled rat, 24h after the 25^th^ PTZ injections (vehicle/PTZ group). (**c**) AFC-5128 pre-treated rat 24h after the 25^th^ PTZ injections (compound/PTZ group). (**d**): Elongated microglia with heavy immunoprecipitation (same magnification, vehicle/PTZ group). Photomicrographs were made by using a light microscope (Axioskop) equipped with a CCD digital camera (AxioCam, ICc 1).

The immunohistochemical studies revealed markedly stronger Iba 1- and GFAP-staining in PTZ-kindled rats in comparison with vehicle-only-treated controls (Fig 7a and 7b, S3a and S3b Fig). A quantitative analysis of the CA3 subfield provided values for Iba 1-staining of 3.3 ± 0.4% in the controls compared to 7.9 ± 1.0 in the PTZ-treated rats and for GFAP-staining values of 3.5 ± 0.5% (controls) to 8.7% ± 0.4% (PTZ-treated rats), respectively. The difference in both cases was highly significant (n = 10; P < 0.01, each, Wilcoxon rank sum test).

Besides the stronger Iba 1-immunostaining in PTZ-kindled rats, microglial cells exhibited a highly branched and ramified morphology, indicating a graded “activated” phenotype. Some scattered microglia showed an elongated formation with larger somata and strong immunoreactivity (Fig 7d). Likewise, astrocytes showed an intensified GFAP-staining with a moderate hypertrophy of cell bodies and highly branched processes in PTZ-kindled rats but no changes to an amoeboid appearance or increasing proliferation with loss of spatial domain organization (see S3b Fig). The number of microglial cells (48.9 ± 1.2 to 49.2 ± 1.5, controls vs. PTZ-samples, calculated per 0.2 mm^2^ each, n = 10) and astrocytes (76.1 ± 4.4 to 82.3 ± 3.0, n = 10), both estimated in the selected CA3 subfield, was not markedly changed. In AFC or JNJ pre-treated animals, immunostaining for both cell markers was clearly reduced compared to vehicle/PTZ-treated animals (Fig 7c, S3c Fig). In P2X7R-immunostained sections, no statistical difference was found between vehicle-only- and vehicle/PTZ-treated animals, in part due to technical difficulties with non-specific background staining. However, in the stratum lucidum in the CA3 region, the field receiving a strong excitatory input from the dentate gyrus via the mossy fibre pathway, PTZ-kindled animals exhibited a striking “P2X7R-like” immunofluorescence that is not observed in the control animals (S4b Fig, top right). However, these results have to be interpreted with caution as the CA3 pre-synaptic/mossy fibre staining we observed is probably not completely reliable with the antibody used.

**Fig 8:**
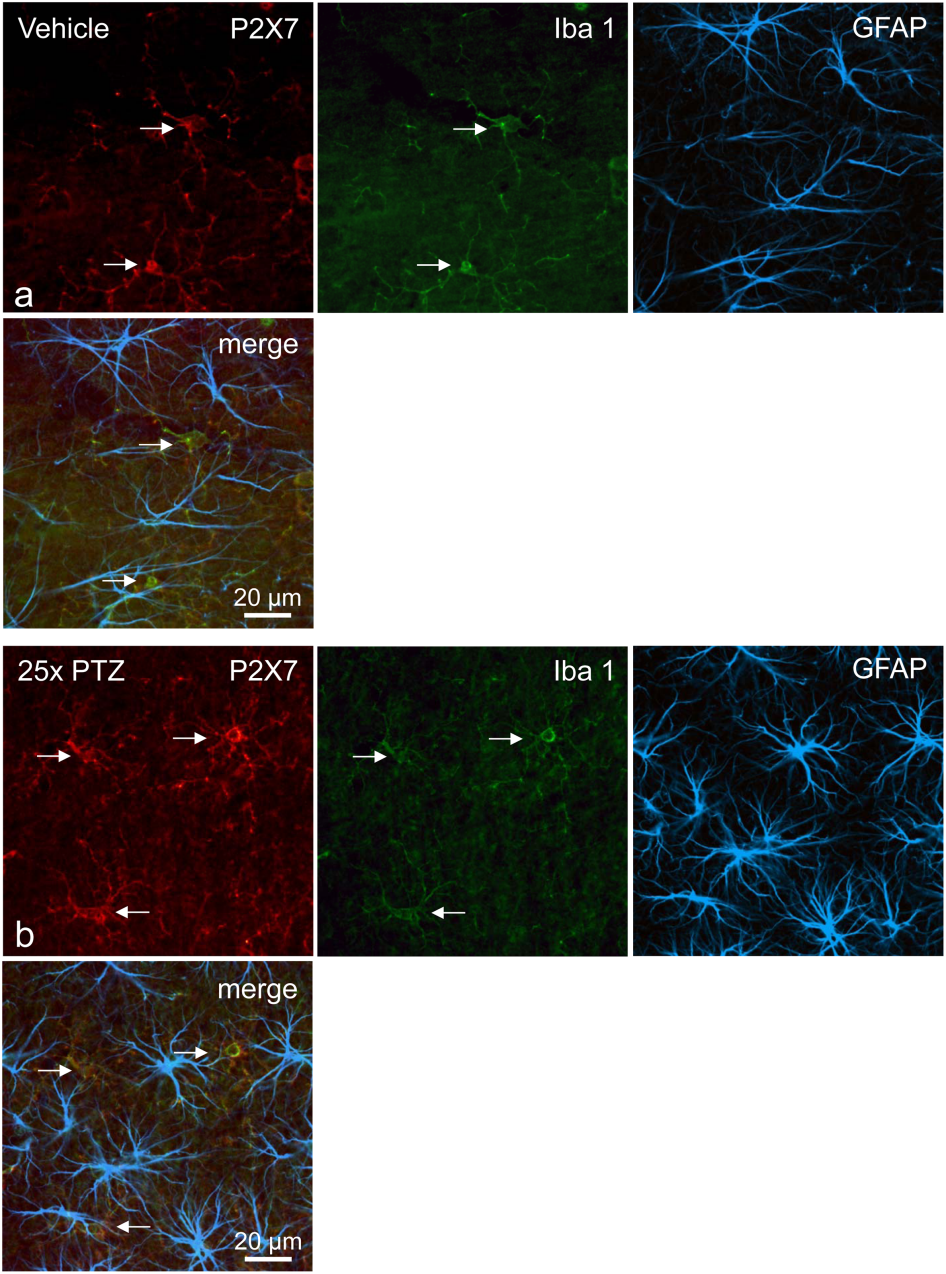
Representative confocal images of triple immunofluorescence showing the localization of P2X7R in the dorsal hippocampus of the rat (CA3 subfield). **(a)** Control animal, 24h after the 25^th^ vehicle injection (20% PEG 400, vehicle-only group), **(b)** PTZ-treated rat, 24h after the 25^th^ PTZ injections (vehicle/PTZ group). Immunofluorescence for P2X7R (Cy3, red), Iba 1 (Cy2, green), GFAP (Alexa Fluor 647-fluorescence, color coded in blue), and the corresponding overlays are presented. Strong P2X7R immunoreactivity was found only on Iba 1-positive microglial cells and their fine elaborated processes (arrows). Photomicrographs were made by using a confocal laser scanning microscope (LSM 510 Meta).

In addition, immunofluorescence studies underlined a marked P2X7R localization on microglia-like cells, but not on astrocytes or neurons in slices of control animals (Fig 8a). Images from PTZ-kindled rats revealed no obvious changes in the colocalisation, e.g. no significant immunofluorescence on astrocytes or neurons (Fig 8b). Strong immunopositive microglia-like cells can frequently be identified. Further studies showed a marked colocalisation of this P2X7R immunofluorescence with synaptophysin, a major membrane glycoprotein of synaptic vesicles, in PTZ-kindled rats (S4d Fig). Other double immunofluorescence studies revealed no clear P2X7R immunostaining of MAP2-positive neurons in the CA3 region both in control and PTZ-kindled rats confirming the findings above (images not shown).

## Discussion

### Aim and Main Findings of This Study

The aim of our study was to investigate possible anticonvulsant and antiepileptogenic effects of four P2X7R blocker (JNJ, AFC, BBG and TIIAS), carefully characterized *in vitro* and in experimental seizure models *in vivo* (information on the compounds used are contained in S1 File “Statement of the Compounds used“). The key outcome of this work was that selected compounds showed synergistic acute anticonvulsant effects in the MES-T and a significant attenuation of the seizure development in the PTZ-kindling model indicating potentially antiepileptogenic effects. In contrast, the P2X7R antagonists alone had limited activity in acute anticonvulsant tests as well as in fully kindled rats. Notably, the effects of JNJ and AFC on kindling development persisted after compound treatment ceased and were associated with reduced glial immunoreactivity. The anti-kindling effect was observed in relation to P2X7R blocking potency, the pharmacokinetic properties and the related brain concentration of the compounds. Altogether, our findings point towards the use of P2X7R antagonists to enhance the anticonvulsant effects of AEDs or to interfere with specific pro-inflammatory pathways, offering novel approaches for the treatment of epilepsy.

The present study represents the first systematic effort to evaluate antagonists of the P2X7R for both acute anticonvulsant effects and for effects in the PTZ-kindling model. The first striking finding here was that none of the four compounds tested were effective in the MES-T test or the PTZ-T test in mice. This indicates that the P2X7R does not contribute to the short lasting seizures triggered by electroshock or a single dose of PTZ in these models. However, in the MES-T test, co-administration of JNJ and AFC with carbamazepine markedly increased the anticonvulsant activity of the AED. This is an interesting finding and supports potential adjunctive use of P2X7R antagonists with conventional AEDs, for example in drug-resistant epilepsy. The mechanisms by which P2X7R antagonism increased the anticonvulsive properties of carbamazepine are unknown. Synergistic effects seem unlikely to be due to pharmacokinetic interactions, as verified for AFC. Animal screening models such as maximal electroshock and PTZ rodent seizure tests are mainly sensitive to drugs blocking Na^+^ and Ca^2+^ channels as well as affecting GABA and glutamate neurotransmission systems [5, 62, 81]. The tested compounds did not have such specific properties or, in the case of BBG, micromolar concentrations were not achieved for efficient Na^+^ channel blockade in the brain (S1 File). The present data contrast with certain previous findings, including apparent anticonvulsant effects of P2X7R antagonists such as BBG in the intra-amygdala KA model of status epilepticus in mice [50, 51, 82]. On the contrary, Kim and Kang [57] reported pro-convulsive properties of P2X7R inhibition in the pilocarpine model of status epilepticus and no effects of P2X7R inhibition on seizures induced by either systemic KA or picrotoxin. There are several reasons which may account for these differences, e.g., chemoconvulsants used and their mechanism of action, induction of status epilepticus or seizure severity, extended neurodegeneration etc. [20, 83].

### PTZ-Kindling Acquisition, Glia Cell Response and P2X7R Blockade

PTZ-induced chemical kindling in rats (or mice) is an accepted model for identifying AEDs and may also detect disease-modifying antiepileptogenic effects [64, 65, 84, 85]. Here we report that P2X7R antagonists attenuate PTZ-induced kindling development in rats, which is in good agreement with a recent study by Amhaoul et al. [56], who showed that treatment of mice suffering from KA-induced epilepsy with the novel P2X7R antagonist JNJ-42253432 reduced the severity of epileptic seizures, although the number of chronic spontaneous recurrent seizures was not altered. Since acute anticonvulsant effects of P2X7R antagonists were not found, it is tempting to speculate that P2X7R antagonism diminishes the progressive seizure development by blocking inflammatory processes in the brain in which the P2X7R is involved. This may include, for example, the activation of microglia and astrocytes with the attendant release of pro-inflammatory cytokines such as IL-1β. P2X7R inhibition may reduce these inflammatory processes as well as gliosis and cell damage that are a key component of the kindling process. Notably, a recent study by Soni et al. [86] showed that BBG alone and in combination with the glutamate transporter GLT-1 up-regulator ceftriaxone significantly decreased the mean kindling score in the PTZ-kindling model in rats, however, drug washout experiments were not performed.

In this regard, our immunohistochemical investigations demonstrated a significant increase both of Iba 1- and GFAP-immunoreactivity after PTZ-kindling acquisition, but no increase in the cell number or proliferation of glial cells in the hippocampal CA3 region, a subfield that, when damaged, has potential ictogenic and epileptogenic potential [87, 88]. No marked morphological signs for gliosis with massive changes to amoeboid forms, as described following status epilepticus in microglial cells [89, 90] were observed in the hippocampus.

It is well-established that microglia has the capacity of a broad spectrum of destructive as well as reparative and neuroprotective functions influencing the delicate balance of pro- and anti-inflammatory signals [91–93]. Also astrocytes as the major glial cell type in the brain, play an important role in complex and essential functions in the CNS. Consequently, astrocytic dysfunction and uncoupling might contribute to a variety of brain disorders and pathologies including epilepsy (for more details, see [94–98]). Thus, we assume that the induced changes in microglia and astrocytes after PTZ-kindling acquisition are likely the result of both ongoing inflammatory processes related to seizure development as well as recovery and compensation processes. However, the complex mechanisms and (patho)physiological consequences of such moderate reactive gliosis resulting in detrimental or beneficial outcomes are still poorly understood [94, 99]. As previously stated, an important finding of our study was that pre-treatment with a P2X7R antagonist (AFC or JNJ) reduced these immunohistochemical responses, indicating that this receptor may play an essential role.

Our triple immunofluorescence images underlined a marked colocalisation of P2X7R-immunoreactivity on Iba 1-positive microglial cells, which was found to be particularly distinctive after the kindling acquisition. Consistent with earlier findings in status epilepticus models, the P2X7R seems to be particularly up-regulated in reactive microglia [43, 48, 89, 100]. As reported before, we observed no significant P2X7R immunofluorescence on astrocytes (comparable with other findings [48, 50]) both in control as well as in PTZ-kindled animals. Although upregulation of *P2rx7* mRNA and protein has been reported after status epilepticus [20], we did not find marked changes in mRNA and protein levels of the P2X7R after PTZ-kindling measured in a restricted number of samples from the whole hippocampus. However, a strong “P2X7R-like” immunoreactivity was noticeably in PTZ-kindled rats in the stratum lucidum of the CA3 region, colocalising with synaptophysin. In good agreement with our results, an increase in P2X7R immunoreactivity was found at mossy fibres in the dentate gyrus and CA3 region of epileptic rats after pilocarpine-induced status epilepticus [46]. Moreover, a strong staining for P2X7R was also described in the stratum lucidum in patients with temporal lobe epilepsy [52]. However, because staining of hippocampal mossy fibre terminals was also found in *P2rx7*^*-/-*^ mice by the identical C-terminal P2X7R antibody, this finding casts doubts concerning the specificity of the P2X7R immunostaining in presynaptic terminals. Thus, again, these data must be interpreted with caution (for further discussion, see [26, 27]).

The results here indicate that blocking the P2X7R is able to delay the development of kindling thereby pointing to antiepileptogenic effects. However, P2X7R antagonists seem insufficient to fully suppress or reverse epileptogenic processes, comparable to some findings of Vezzani’s group with other anti-inflammatory tools [18, 39, 101]. This is not altogether surprising since the pathophysiology of epileptic disorders is highly complex, involving numerous disturbances in neurotransmission systems, receptors, ion channels, microglial and astrocytic function as well as genetic causes [102]; P2X7R signalling is only one of the multiple pathways activated. Moreover, brain inflammation mediated by P2X7R may have a bi-functional role, initially more protective but detrimental after an excessive or chronic status [32]. Additionally, important physiological functions of P2X7R must be taken into account, e.g. the stimulation of glutamate and GABA release from astrocytes, as well as microglial release of neurotrophic factors that may be inhibited by P2X7R blockade [103]. Thus, the role of P2X7R might change during the course of the same disease. Complete inhibition of P2X7R with too high doses, therefore, might have an unfavourable outcome. Consequently, the question of optimal time and dosing regimen for intervention with P2X7R blocker as well as potential drug combinations needs to be resolved.

### Conclusion and Outlook

In conclusion, our study indicates that P2X7R blockade has a potential antiepileptogenic effect in the PTZ-kindling model in rats. Treatment with the novel P2X7R antagonists AFC and JNJ significantly delayed the development of seizures and inhibited glial inflammatory responses. However, these compounds did not completely prevent an increase in seizure susceptibility to PTZ during the kindling procedure. On the other hand, P2X7R blocker alone exhibited no anticonvulsant activity in traditional screening tests, such as the MES- and PTZ seizure threshold test. Nevertheless, in conjunction with an anticonvulsant drug the electroconvulsive threshold was significantly increased in the MES-T. We conclude that P2X7R inhibition exerts a protective effect against kindling development, but a single-target strategy might be insufficient to suppress epileptogenesis. Multiple-target treatments that act on different pathways involved in the complex epileptogenic processes may be of prospective interest. Further research should explore the possible antiepileptogenic and/or neuroprotective potential of other P2X7R antagonists and additional P2 receptor subtypes, their interplay with anti-inflammatory substances as well as multi-drug (multi-target) combinations alone and in co-medication with AEDs in diverse epilepsy models including models of drug-resistant seizures.

## Supporting Information

S1 Fig Modulation of ATP-induced [Ca^2+^]_i_ response (Ca^2+^ entry traces corresponding to Fig 2a and 2b). The studies were carried out in HEK293 cells, stably transfected with (**a**) mouse P2X7R and (**b**) rat P2X7R, respectively (fluo-4 microfluorometry, cell suspension, microplate reader). Compounds and cells were placed in a 384-well microtitre plate. After 10 base-line cycles, ATP (final 1 mM) was injected into each well. The time course of recorded fluorescence intensities is given in arbitrary units (a.u.). Representative recordings demonstrate the concentration-dependent blocking by the four tested compounds tanshinone IIA-SO_3_Na (TIIAS, red), Brilliant Blue G (BBG, blue), AFC-5128 (AFC, green), JNJ-47965567 (JNJ, yellow). On the right: concentrations (in μM). Control recordings (C, black) with solvent HBS/DMSO.
(PDF)

S2 Fig Influence of the PTZ-kindling on P2X7R messenger RNA and protein level. **(a)** *P2rx7* messenger RNA levels in whole hippocampus in the PTZ-kindling model. Rats were examined 24h after the 10^th^ or 25^th^ injection of PTZ; untreated (naïve) and saline (NaCl)-treated rats served as controls. Data were normalized to expression of β-actin and represented as relative quantity (RQ) values. **(b)** Representative Western blots (n = 1 per lane) and graphs from whole hippocampus showing no changes of the P2X7R protein level after the 10^th^ PTZ injection and a tendency for a small increase in fully kindled rats after the 25^th^ PTZ injection in comparison with saline-treated rats as controls (n = 4 per group, each). Data were normalized to expression of β-actin and represented as RQ values.
(PDF)

S3 Fig Representative light microscopy photomicrographs showing GFAP-immunopositive astrocytes from the right dorsal anterior hippocampus of the rat. (**a**) Control animal, 24h after the 25^th^ vehicle injection (20% PEG 400, vehicle-only group); higher magnification view of CA3 subfield (outlined area) on the right. (**b**) PTZ-kindled rat, 24h after the 25^th^ PTZ injections (vehicle/PTZ group). (**c**) JNJ-47965567 pre-treated rat 24h after the 25^th^ PTZ injections (compound/PTZ group).
(PDF)

S4 Fig Representative confocal images of double immunofluorescence showing the colocalisation of P2X7R and synaptophysin (GFAP) in the CA3 subfield. The CA3 overview images (above) show double immunofluorescence for P2X7R (Cy3, red) and GFAP (Cy2, green), respectively. **(a)** Control animal (vehicle-only group), **(b)** PTZ-treated rat (vehicle/PTZ group). A striking “P2X7R-like” immunofluorescence in the PTZ-treated rat can be observed in the stratum lucidum (sl) above the cell bodies of CA3 pyramidal cells (p). In order to present more clearly the P2X7R immunoreactivity, GFAP immunofluorescence was not shown in the PTZ-treated rat. **(c)** Control animal (vehicle-only group), **(d)** PTZ-treated rat (vehicle/PTZ group). High-power view of the stratum lucidum showing colocalisation of P2X7R (Cy3, red) and synaptophysin (Cy2, green) immunofluorescence (merging to yellow), frequently found in the PTZ-treated rat (some examples are marked by small arrows). Small black holes correspond to dendrites of pyramidal cells.
(PDF)

S1 File Short statement on the compounds used. (DOCX)
