# Supplementary figures and images for: Critical Evaluation of P2X7 Receptor Antagonists in Selected Seizure Models
Source: PLoS One. 2016 Jun 9;11(6):e0156468. doi: 10.1371/journal.pone.0156468 (PMC4900628; doi:10.1371/journal.pone.0156468)

a

## Mouse P2X7R

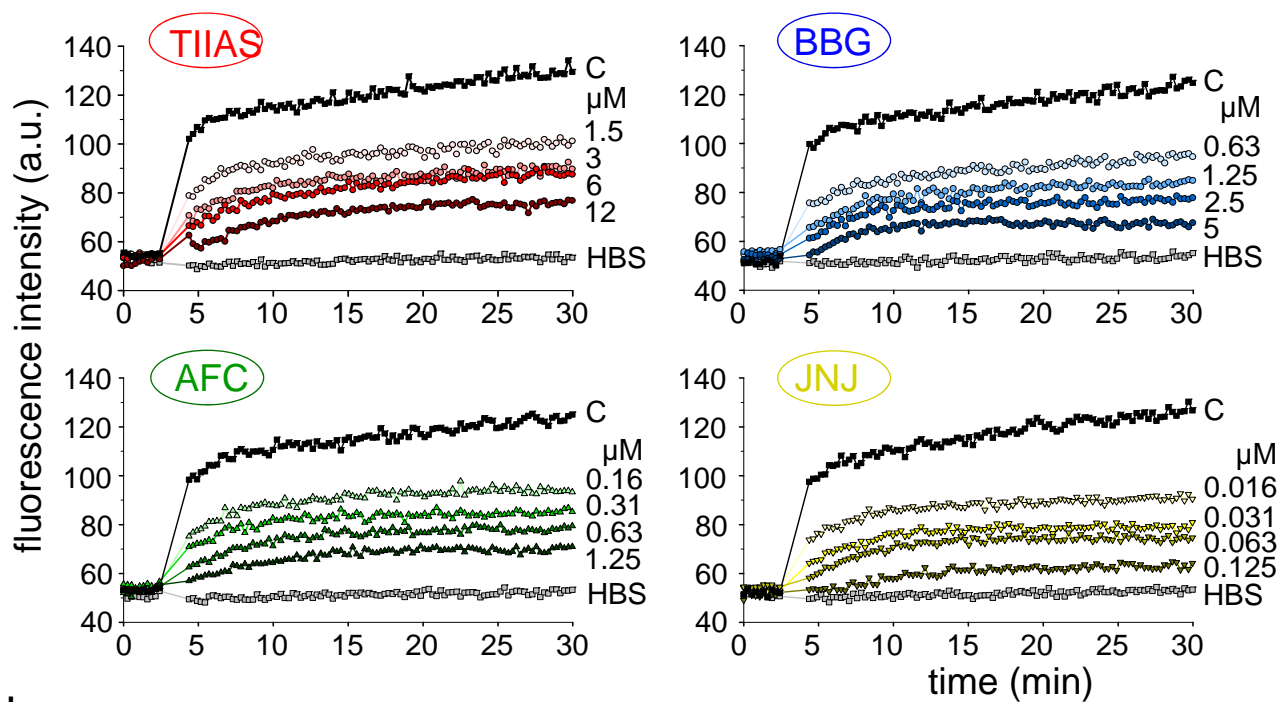

b

## Rat P2X7R

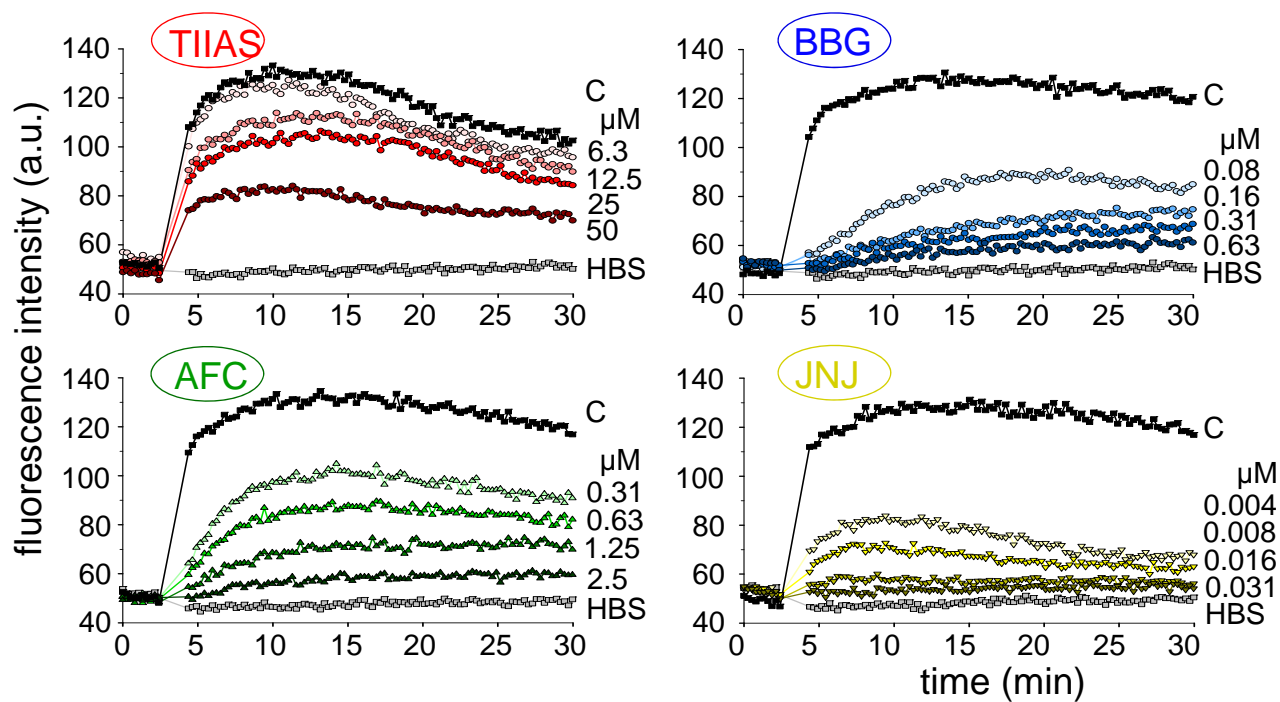

Supplement: S1 Fig — The studies were carried out in HEK293 cells, stably transfected with (a) mouse P2X7R and (b) rat P2X7R, respectively (fluo-4 microfluorometry, cell suspension, microplate reader). Compounds and cells were placed in a 384-well microtitre plate. After 10 base-line cycles, ATP (final 1 mM) was injected into each well. The time course of recorded fluorescence intensities is given in arbitrary units (a.u.). Representative recordings demonstrate the concentration-dependent blocking by the four tested compounds tanshinone IIA-SO3Na (TIIAS, red), Brilliant Blue G (BBG, blue), AFC-5128 (AFC, green), JNJ-47965567 (JNJ, yellow). On the right: concentrations (in μM). Control recordings (C, black) with solvent HBS/DMSO. (PDF) [file pone.0156468.s001.pdf]

a

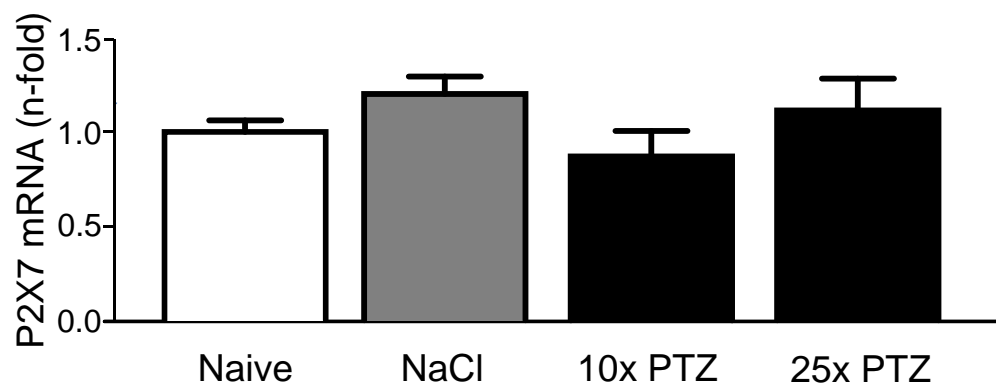

b

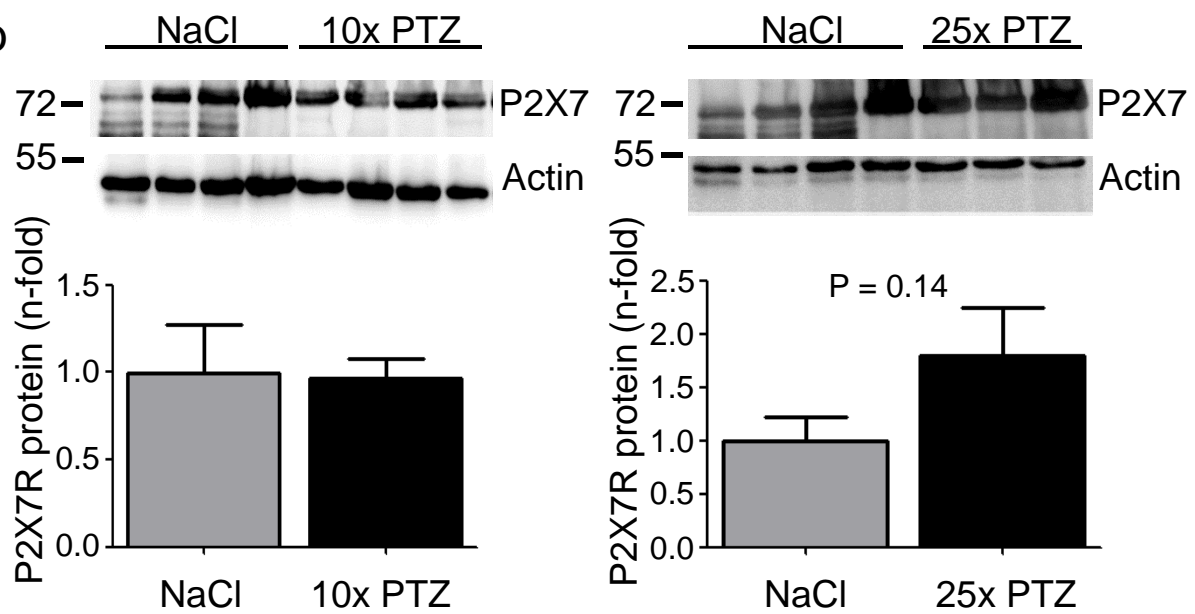

Supplement: S2 Fig — (a) P2rx7 messenger RNA levels in whole hippocampus in the PTZ-kindling model. Rats were examined 24h after the 10th or 25th injection of PTZ; untreated (naïve) and saline (NaCl)-treated rats served as controls. Data were normalized to expression of β-actin and represented as relative quantity (RQ) values. (b) Representative Western blots (n = 1 per lane) and graphs from whole hippocampus showing no changes of the P2X7R protein level after the 10th PTZ injection and a tendency for a small increase in fully kindled rats after the 25th PTZ injection in comparison with saline-treated rats as controls (n = 4 per group, each). Data were normalized to expression of β-actin and represented as RQ values. (PDF) [file pone.0156468.s002.pdf]

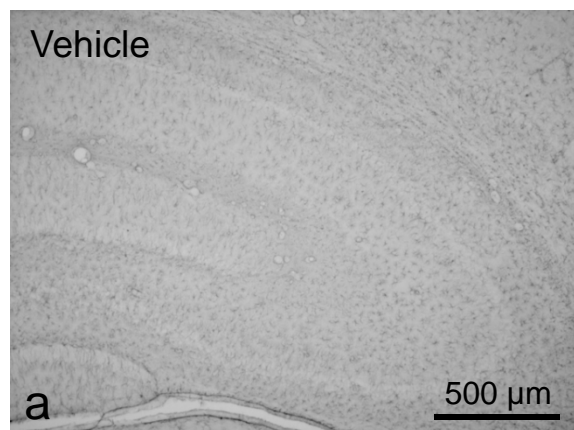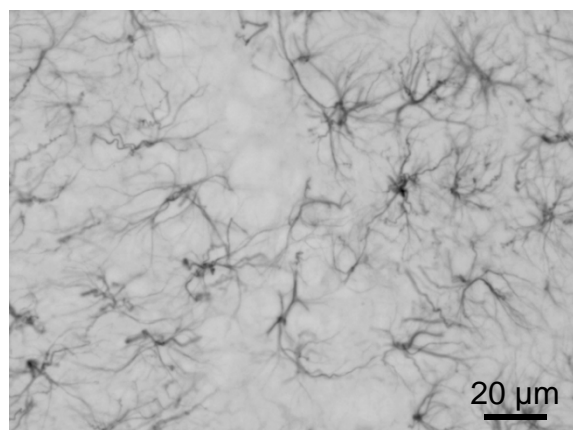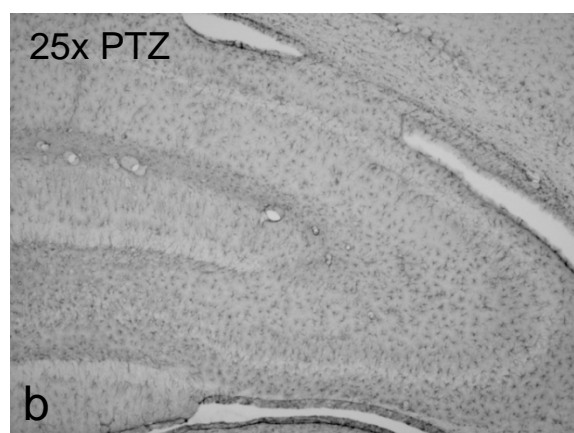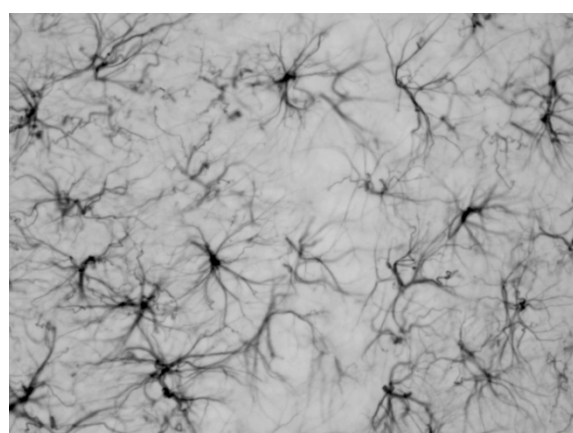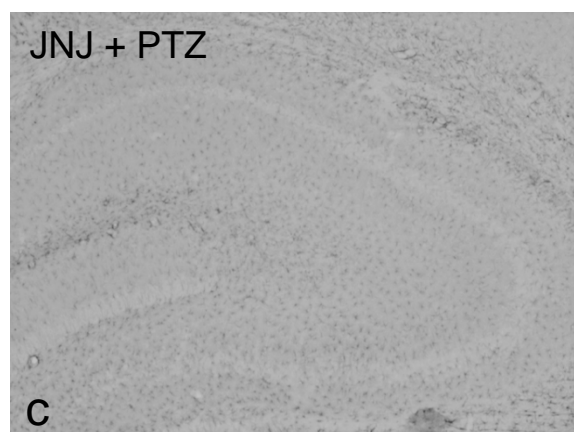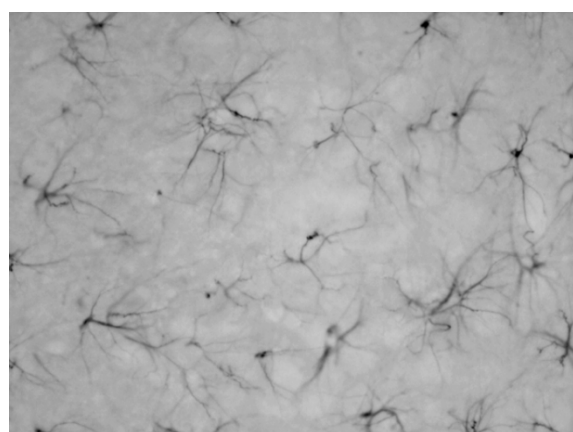

Supplement: S3 Fig — (a) Control animal, 24h after the 25th vehicle injection (20% PEG 400, vehicle-only group); higher magnification view of CA3 subfield (outlined area) on the right. (b) PTZ-kindled rat, 24h after the 25th PTZ injections (vehicle/PTZ group). (c) JNJ-47965567 pre-treated rat 24h after the 25th PTZ injections (compound/PTZ group). (PDF) [file pone.0156468.s003.pdf]

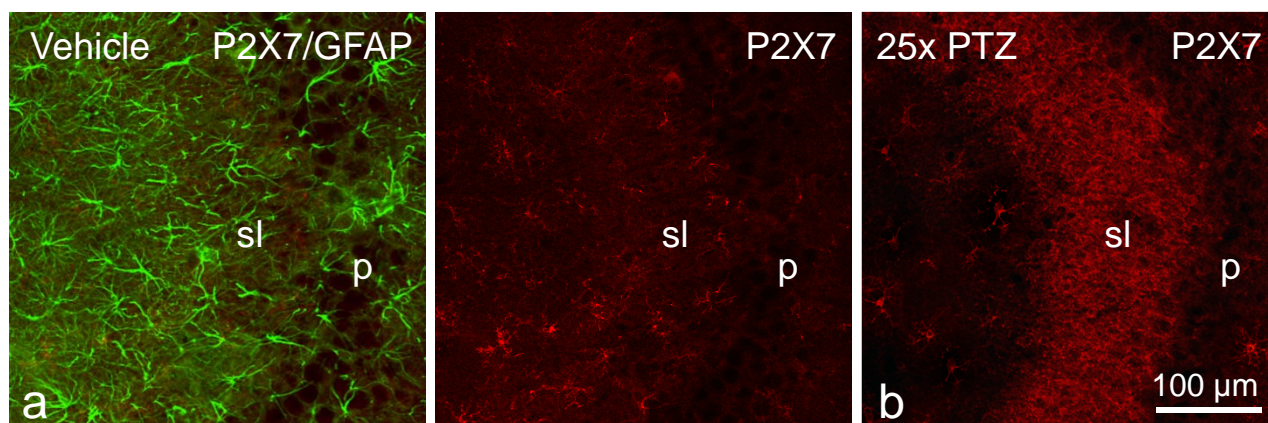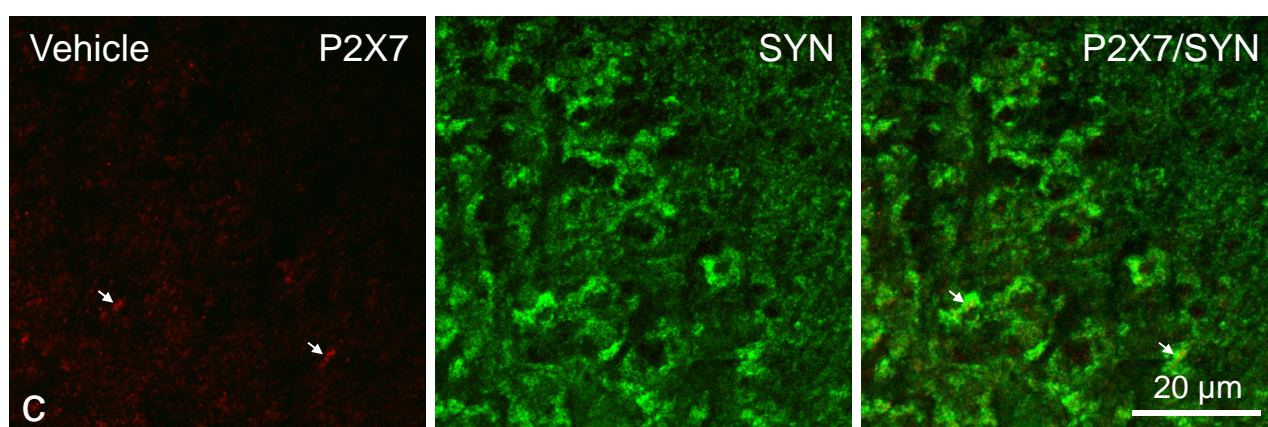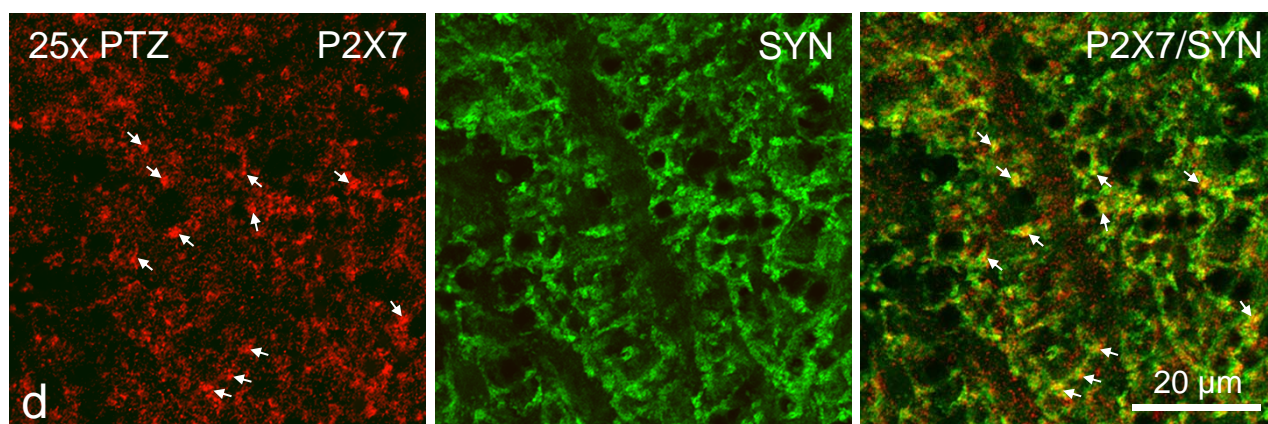

Supplement: S4 Fig — The CA3 overview images (above) show double immunofluorescence for P2X7R (Cy3, red) and GFAP (Cy2, green), respectively. (a) Control animal (vehicle-only group), (b) PTZ-treated rat (vehicle/PTZ group). A striking “P2X7R-like” immunofluorescence in the PTZ-treated rat can be observed in the stratum lucidum (sl) above the cell bodies of CA3 pyramidal cells (p). In order to present more clearly the P2X7R immunoreactivity, GFAP immunofluorescence was not shown in the PTZ-treated rat. (c) Control animal (vehicle-only group), (d) PTZ-treated rat (vehicle/PTZ group). High-power view of the stratum lucidum showing colocalisation of P2X7R (Cy3, red) and synaptophysin (Cy2, green) immunofluorescence (merging to yellow), frequently found in the PTZ-treated rat (some examples are marked by small arrows). Small black holes correspond to dendrites of pyramidal cells. (PDF) [file pone.0156468.s004.pdf]
