# Supplementary material for: Critical Evaluation of P2X7 Receptor Antagonists in Selected Seizure Models
Source: PLoS One. 2016 Jun 9;11(6):e0156468. doi: 10.1371/journal.pone.0156468 (PMC4900628; doi:10.1371/journal.pone.0156468)
Supplement: S1 File — (DOCX) [file pone.0156468.s005.docx]

**Supporting Information**

**Critical Evaluation of P2X7 Receptor Antagonists**

**in Selected Seizure Models**

Wolfgang Fischer, Heike Franke, Ute Krügel, Heiko Müller, Klaus Dinkel,

Brian Lord, Michael A. Letavic, David C. Henshall, Tobias Engel

**Short statement on the compounds used**

Initial studies using a fluorometric Ca^2+^ assay and different HEK293 cell lines stably expressing human, rat and mouse P2X7R served to characterize and compare the potency and the species specificity of the four selected compounds, tested in various experimental seizure models.

JNJ (JNJ-47965567), a novel P2X7R antagonist, potently blocked the rat and mouse P2X7R at low nanomolar concentrations, even an order of magnitude higher than the potent non-competitive antagonist AZ-10606120 [1] used as reference substance (Fig 2b, a; Table 1; for illustration of Ca^2+^ entry traces see also S1 Fig). JNJ, a phenylpiprazine derivative was described as centrally permeable, high-affinity, selective P2X7R antagonist [2, 3]. Moreover, it blocked the BzATP-induced release of the pro-inflammatory cytokine IL-1β in rat microglia and exhibited efficacy in animal models of neuropathic pain [3]. In consequence, this compound was suggested as a useful tool for the examination of the role of P2X7R in CNS pathophysiology. In the present study, after a single dose of JNJ (15 mg/kg s.c.), rat brain concentrations of 5 µM were achieved 30 min after injection, which were 1000-fold higher than that required for 50% inhibition of Ca^2+^ influx (IC_50_ = 3 nM) in the fluorometric assay (Fig 2b; Table 1). In addition, the determined P2X7R occupancy was 98%. Thus, it can be assumed that the P2X7R was almost completely blocked at this doses.

AFC (AFC-5128) is also a novel P2X7R antagonist that showed a similar potency to the known competitive P2X7R antagonist A-438079 [4] at the two rodent P2X7Rs, but has a potency one to two orders of magnitude lower than JNJ (Fig 2a, b). The potency of AFC for the human P2X7R was much higher and in the same order of JNJ and AZ-100606120, respectively (Fig 2c; Table 1). AFC, an N-indol-3-carboxamide derivative, is a centrally permeable, potent and selective P2X7R antagonist [5] (following information on compound characteristics provided by Affectis Pharmaceuticals, Dortmund). Repeated doses up to 500 mg/kg twice a day p.o. are well tolerated in rats. Moreover, this compound inhibited the ATP-triggered release of IL-1ß from lipopolysaccharide (LPS)-primed human macrophages and THP1 human monocyte cells in nanomolar (30-50 nM) concentrations. AFC showed strong anti-inflammatory and analgesic effects in animal models of multiple sclerosis, neuropathic and inflammatory pain. Lead optimization and preclinical development studies are in progress. In the present study, following a single dose of 30 mg/kg i.p., rat brain concentrations of 15 µM were achieved 45 min after injection, which were 30-fold higher than the IC_50_ value of 0.5 µM estimated in our fluorometric Ca^2+^ assay (see Fig 2b; Table 1). This may result in an approximately 80% inhibition of the P2X7R.

BBG (Brilliant Blue G), a polysulfonated dye, is a potent, non-competitive P2X7R antagonist in nanomolar concentrations [6], but displaying also blocking effects against other P2X receptors such as P2X4 and P2X5 with micromolar efficacy [7]. Moreover, BBG can cross the blood-brain barrier, has low toxicity, proved to be neuroprotective and revealed therapeutic potential in several animal models of neurodegenerative diseases, e.g., Alzheimer’s disease, Huntington’s disease, traumatic brain injury or global cerebral ischemia [8-12]. It has been shown that this compound attenuates LPS-mediated microglial activation and has anti-inflammatory and antidepressant effects in mice after LPS-administration [13, 14]. Of interest, BBG was found to inhibit voltage-gated Na^+^ channels in neuroblastoma cells in micromolar concentrations [15]. Therefore, BBG was used as an alternative for our studies as it was commercially available in larger amounts. As previously shown, BBG revealed high potency for the rat P2X7R comparable to that of AZ-10606120 (Fig 2b, Table 1) and weaker effectiveness for mouse and human P2X7R (Fig 2a, and 2c, Table 1). However, the dark blue colour of higher concentrated BBG solutions may influence the fluo-4 fluorometric measurements and therefore the determined IC_50_ values must be regarded with caution in our assay. In the present study, the chosen dose of 50 mg/kg i.p. yielded rat brain concentrations of 20 nM 45 min after injection with a very low brain-plasma ratio of 0.01. In contrast, higher brain concentrations of 150 nM were reported in mice after 45.5 mg/kg i.p. (administration every 48h for 1 month) by spectrophotometric analysis, but the brain-plasma ratio was with 0.02 also very low [11]. In the fluorometric Ca^2+^ assay, 20 nM would correspond to an approximately 30% inhibition of the ATP-induced Ca^2+^ response (Fig 2b). Thus, it is possible that the achieved brain concentrations are too low to suppress effectively P2X7R signalling.

TIIAS (tanshinone IIA sulphonic sodium) is a water-soluble derivative of the natural compound tanshinone IIA and is used for treating cardiovascular diseases in China [16]. Continuing studies revealed cardioprotective, antioxidant, anti-dyslipidemia as well as anti-inflammatory properties [17, 18]. On the other hand, the natural compound tanshinone IIA, extracted from the root of red sage (*Salvia miltiorrhiza*), is a diterpenoid quinone and a major bioactive constituent of “Danshen”, a traditional Chinese herbal medicine with therapeutic application for the treatment of cardiovascular and other diseases [18-21]. Moreover, remarkable anti-inflammatory and neuroprotective effects of tanshinone IIA were demonstrated in focal cerebral ischemia models or after traumatic spinal cord injury [22-24]. It has been recently shown that TIIAS, but not tanshinone IIA, acts as a potent non-competitive inhibitor of the human P2X7R, possibly by binding to the intracellular domain of the receptor [25]. Notably, this study also demonstrated that this compound inhibited the ATP-triggered release of IL-1ß from LPS-primed human macrophages indicating anti-inflammatory properties. The blocking potency for mouse and rat P2X7R was much lower with IC_50_ values of ≥6 and ≥30 µM, respectively, re-evaluated under the present experimental condition (Fig 2a and b; Table 1). Moreover, our results revealed that TIIAS has the potential to cross the blood-brain barrier, but a single dose of 30 mg/kg i.p. yielded rat brain concentrations of only 500 nM after 45 min corresponding to the low brain-plasma ratio of 0.02. At this brain level, only a small blocking effect of P2X7R can be expected (Fig 2a and b). Together, it is conceivably that TIIAS possesses interesting beneficial effects like the natural compound tanshinone IIA. Thus, we decided to include TIIAS, which was also commercially available in larger amounts, in our studies. However, further improvements in potency and pharmacokinetic properties would still be desirable in the future [16]. Of note, a composite formula with TIIAS has completed Phase II clinical trials in US involving patients with chronic stable angina pectoris (for details, see [19].

**References**

1. Michel AD, Chambers LJ, Walter DS (2008) [Negative and positive allosteric modulators of the P2X_7_ receptor.](http://www.ncbi.nlm.nih.gov/pubmed/18071294) Br J Pharmacol 53(4):737-750.
2. Letavic MA, Lord B, Bischoff F, Hawryluk NA, Pieters S, Rech JC, Sales Z, Velter AI, Ao H, Bonaventure P, Contreras V, Jiang X, Morton KL, Scott B, Wang Q, Wickenden AD, Carruthers NI, Bhattacharya A (2013) [Synthesis and pharmacological characterization of two novel, brain penetrating P2X7 antagonists.](http://www.ncbi.nlm.nih.gov/pubmed/24900687) ACS Med Chem Lett 4(4):419-422.
3. Bhattacharya A, Wang Q, Ao H, Shoblock JR, Lord B, Aluisio L, Fraser I, Nepomuceno D, Neff RA, Welty N, Lovenberg TW, Bonaventure P, Wickenden AD, Letavic MA (2013) [Pharmacological characterization of a novel centrally permeable P2X7 receptor antagonist: JNJ-47965567.](http://www.ncbi.nlm.nih.gov/pubmed/23889535) Br J Pharmacol 170(3):624-640.
4. Nelson DW, Sarris K, Kalvin DM, Namovic MT, Grayson G, Donnelly-Roberts DL, Harris R, Honore P, Jarvis MF, Faltynek CR, Carroll WA (2008) [Structure-activity relationship studies on N'-aryl carbohydrazide P2X7 antagonists.](http://www.ncbi.nlm.nih.gov/pubmed/18438986) J Med Chem 51(10):3030-3044.
5. Bös M (2009) Inventor; Affectis Pharmaceuticals AG, assignee. Novel PX7 antagonists and their use. Patent WO 2009118175A1.
6. Jiang LH, Mackenzie AB, North RA, Surprenant A (2000) [Brilliant blue G selectively blocks ATP-gated rat P2X_7_ receptors.](http://www.ncbi.nlm.nih.gov/pubmed/10860929) Mol Pharmacol 58(1):82-88.
7. Coddou C, Yan Z, Obsil T, Huidobro-Toro JP, Stojilkovic SS (2011) [Activation and regulation of purinergic P2X receptor channels.](http://www.ncbi.nlm.nih.gov/pubmed/21737531) Pharmacol Rev 63(3): 641-683.
8. Peng W, Cotrina ML, Han X, Yu H, Bekar L, Blum L, Takano T, Tian GF, Goldman SA, Nedergaard M (2009) [Systemic administration of an antagonist of the ATP-sensitive receptor P2X7 improves recovery after spinal cord injury.](http://www.ncbi.nlm.nih.gov/pubmed/19666625) Proc Natl Acad Sci U S A 106(30):12489-12493.
9. Takenouchi T, Sekiyama K, Sekigawa A, Fujita M, Waragai M, Sugama S, Iwamaru Y, Kitani H, Hashimoto M (2010) [P2X7 receptor signaling pathway as a therapeutic target for neurodegenerative diseases.](http://www.ncbi.nlm.nih.gov/pubmed/20143170) Arch Immunol Ther Exp (Warsz) 58(2):91-96.
10. [Arbeloa J](http://www.ncbi.nlm.nih.gov/pubmed?term=Arbeloa%20J%5BAuthor%5D&cauthor=true&cauthor_uid=22186422), [Pérez-Samartín A](http://www.ncbi.nlm.nih.gov/pubmed?term=P%C3%A9rez-Samart%C3%ADn%20A%5BAuthor%5D&cauthor=true&cauthor_uid=22186422), [Gottlieb M](http://www.ncbi.nlm.nih.gov/pubmed?term=Gottlieb%20M%5BAuthor%5D&cauthor=true&cauthor_uid=22186422), [Matute C](http://www.ncbi.nlm.nih.gov/pubmed?term=Matute%20C%5BAuthor%5D&cauthor=true&cauthor_uid=22186422) (2012) P2X7 receptor blockade prevents ATP excitotoxicity in neurons and reduces brain damage after ischemia. [Neurobiol Dis](http://www.ncbi.nlm.nih.gov/pubmed/22186422##) 45(3):954-961.
11. Diaz-Hernandez JI, Gomez-Villafuertes R, León-Otegui M, Hontecillas-Prieto L, Del Puerto A, Trejo JL, Lucas JJ, Garrido JJ, Gualix J, Miras-Portugal MT, Diaz-Hernandez M (2012) [In vivo P2X7 inhibition reduces amyloid plaques in Alzheimer's disease through GSK3β and secretases.](http://www.ncbi.nlm.nih.gov/pubmed/22048123) Neurobiol Aging 33(8):1816-1828.
12. Yu Q, Guo Z, Liu X, Ouyang Q, He C, Burnstock G, Yuan H, Xiang Z (2013) [Block of P2X7 receptors could partly reverse the delayed neuronal death in area CA1 of the hippocampus after transient global cerebral ischemia.](http://www.ncbi.nlm.nih.gov/pubmed/23877788) Purinergic Signal 9(4):663-675.
13. Lu K, Wang J, Hu B, Shi X, Zhou J, Tang Y, Peng Y (2013) [Brilliant blue G attenuates lipopolysaccharide-mediated microglial activation and inflammation.](http://www.ncbi.nlm.nih.gov/pubmed/25206704) Neural Regen Res 8(7):599-608.
14. Ma M, Ren Q, Zhang JC, Hashimoto K (2014) [Effects of Brilliant Blue G on Serum Tumor Necrosis Factor-α Levels and Depression-like Behavior in Mice after Lipopolysaccharide Administration.](http://www.ncbi.nlm.nih.gov/pubmed/24851118) Clin Psychopharmacol Neurosci 12(1):31-36.
15. Jo S, Bean BP (2011) [Inhibition of neuronal voltage-gated sodium channels by brilliant blue G.](http://www.ncbi.nlm.nih.gov/pubmed/21536754) Mol Pharmacol 80(2):247-257.
16. [Tian XH](http://www.ncbi.nlm.nih.gov/pubmed?term=Tian%20XH%5BAuthor%5D&cauthor=true&cauthor_uid=23094864), [Wu JH](http://www.ncbi.nlm.nih.gov/pubmed?term=Wu%20JH%5BAuthor%5D&cauthor=true&cauthor_uid=23094864) (2013) Tanshinone derivatives: a patent review (January 2006 - September 2012). [Expert Opin Ther Pat](http://www.ncbi.nlm.nih.gov/pubmed/?term=Tian+XH+tanshinone##) 23(1):19-29 [ASN Neuro.](http://www.ncbi.nlm.nih.gov/pubmed/22339481) 2012 Apr 5;4(3). pii: e00082. doi: 10.1042/AN20120010.
17. Yang R, Liu A, Ma X, Li L, Su D, Liu J (2008) [Sodium tanshinone IIA sulfonate protects cardiomyocytes against oxidative stress-mediated apoptosis through inhibiting JNK activation.](http://www.ncbi.nlm.nih.gov/pubmed/18427283) J Cardiovasc Pharmacol 51(4):396-401.
18. Wei B, You MG, Ling JJ, Wei LL, Wang K, Li WW, Chen T, Du QM, Ji H (2013) [Regulation of antioxidant system, lipids and fatty acid β-oxidation contributes to the cardioprotective effect of sodium tanshinone IIA sulphonate in isoproterenol-induced myocardial infarction in rats.](http://www.ncbi.nlm.nih.gov/pubmed/23958267) Atherosclerosis. 230(1):148-156
19. [Gao S](http://www.ncbi.nlm.nih.gov/pubmed?term=Gao%20S%5BAuthor%5D&cauthor=true&cauthor_uid=21774934), [Liu Z](http://www.ncbi.nlm.nih.gov/pubmed?term=Liu%20Z%5BAuthor%5D&cauthor=true&cauthor_uid=21774934), [Li H](http://www.ncbi.nlm.nih.gov/pubmed?term=Li%20H%5BAuthor%5D&cauthor=true&cauthor_uid=21774934), [Little PJ](http://www.ncbi.nlm.nih.gov/pubmed?term=Little%20PJ%5BAuthor%5D&cauthor=true&cauthor_uid=21774934), [Liu P](http://www.ncbi.nlm.nih.gov/pubmed?term=Liu%20P%5BAuthor%5D&cauthor=true&cauthor_uid=21774934), [Xu S](http://www.ncbi.nlm.nih.gov/pubmed?term=Xu%20S%5BAuthor%5D&cauthor=true&cauthor_uid=21774934) (2012) Cardiovascular actions and therapeutic potential of tanshinone IIA. [Atherosclerosis](http://www.ncbi.nlm.nih.gov/pubmed/21774934##) 220(1):3-10
20. [Shang Q](http://www.ncbi.nlm.nih.gov/pubmed/?term=Shang%20Q%5BAuthor%5D&cauthor=true&cauthor_uid=22454677), [Xu H](http://www.ncbi.nlm.nih.gov/pubmed/?term=Xu%20H%5BAuthor%5D&cauthor=true&cauthor_uid=22454677), [Huang L](http://www.ncbi.nlm.nih.gov/pubmed/?term=Huang%20L%5BAuthor%5D&cauthor=true&cauthor_uid=22454677) (2012) Tanshinone IIA: A Promising Natural Cardioprotective Agent. [Evid Based Complement Alternat Med](http://www.ncbi.nlm.nih.gov/pubmed/22454677) 2012:716459. doi: 10.1155/2012/716459.
21. Xu S, Liu P (2013) [Tanshinone II-A: new perspectives for old remedies.](http://www.ncbi.nlm.nih.gov/pubmed/23231009) Expert Opin Ther Pat 23(2):149-153
22. [Dong K](http://www.ncbi.nlm.nih.gov/pubmed/?term=Dong%20K%5BAuthor%5D&cauthor=true&cauthor_uid=18844253), [Xu W](http://www.ncbi.nlm.nih.gov/pubmed/?term=Xu%20W%5BAuthor%5D&cauthor=true&cauthor_uid=18844253), [Yang J](http://www.ncbi.nlm.nih.gov/pubmed/?term=Yang%20J%5BAuthor%5D&cauthor=true&cauthor_uid=18844253), [Qiao H](http://www.ncbi.nlm.nih.gov/pubmed/?term=Qiao%20H%5BAuthor%5D&cauthor=true&cauthor_uid=18844253), [Wu L](http://www.ncbi.nlm.nih.gov/pubmed/?term=Wu%20L%5BAuthor%5D&cauthor=true&cauthor_uid=18844253) (2009) Neuroprotective effects of Tanshinone IIA on permanent focal cerebral ischemia in mice. [Phytother Res](http://www.ncbi.nlm.nih.gov/pubmed/?term=Dong+K+tanshinone) 23(5):608-613.
23. Wang L, Zhang X, Liu L, Cui L, Yang R, Li M, Du W (2010) [Tanshinone II A down-regulates HMGB1, RAGE, TLR4, NF-kappaB expression, ameliorates BBB permeability and endothelial cell function, and protects rat brains against focal ischemia.](http://www.ncbi.nlm.nih.gov/pubmed/20043889) Brain Res 1321:143-151.
24. Yin X, Yin Y, Cao FL, Chen YF, Peng Y, Hou WG, Sun SK, Luo ZJ (2012) [Tanshinone IIA attenuates the inflammatory response and apoptosis after traumatic injury of the spinal cord in adult rats.](http://www.ncbi.nlm.nih.gov/pubmed/22675554) PLoS One 7(6):e38381. doi: 10.1371/journal.pone.0038381.
25. Kaiser M, Sobottka H, Fischer W, Schaefer M, Nörenberg W (2014) [Tanshinone II A sulfonate, but not tanshinone II A, acts as potent negative allosteric modulator of the human purinergic receptor P2X7.](http://www.ncbi.nlm.nih.gov/pubmed/24970925) J Pharmacol Exp Ther 350(3):531-542.
